# Comprehensive analysis of the RBP regulome reveals functional modules and drug candidates in liver cancer

**DOI:** 10.1038/s41598-026-58864-6

**Published:** 2026-06-26

**Authors:** Mateusz Garbulowski, Riccardo Mosca, Carlos J. Gallardo-Dodd, Claudia Kutter, Erik L. L. Sonnhammer

**Affiliations:** 1 Science for Life Laboratory, Department of Biochemistry and Biophysics, Stockholm University, Solna, Sweden; 2 Department of Immunology, Genetics and Pathology, Uppsala University, Uppsala, Sweden; 3 Science for Life Laboratory, Department of Microbiology, Tumor, and Cell Biology, Karolinska Institute, Solna, Sweden

**Keywords:** Liver cancer, Gene regulatory networks, Bioinformatics, Gene expression analysis

## Abstract

RNA binding proteins (RBPs) are essential components of the transcriptomic regulome. Understanding the RBP regulome in cancer cells is crucial for uncovering carcinogenesis mechanisms and identifying novel therapeutic targets. In this study, we aimed to reveal the regulome of liver cancer upon specific perturbations. To achieve this, we applied a consensus Gene Regulatory Network (GRN) approach using knockdown data focusing on the liver cancer cell line HepG2. By integrating multiple GRNs inferred from diverse computational methods, we constructed a comprehensive regulatory network. To validate our findings, we evaluated the consensus GRN by focusing on characterizing key regulatory interactions in liver cancer. We used eCLIP-seq and RAP-seq data to verify RBP interactions and binding sites. In addition, we performed an enrichment analysis of network modules and in silico drug repurposing based on the inferred GRN. Taken together, our findings highlight the critical role of RBP-mediated regulation in liver cancer, which can be used to improve treatment strategies and develop further research.

**Supplementary Information:**

The online version contains supplementary material available at 10.1038/s41598-026-58864-6.

## Introduction

RNA binding proteins (RBPs) play a crucial role in regulating gene expression by influencing a broad range of post-transcriptional processes. These processes include RNA decay, splicing, stabilization, translation, and transport (Fig. S1A). RBPs interact with RNA targets through various mechanisms, most principally via well-characterized RNA-binding domains (RBDs), often referred to as canonical RBPs^1^. Studies on the role of RBPs in cancer imply that they contribute to mechanisms of progression, metastasis, and drug resistance^2–7^. For instance, a recent study has highlighted the role of RBPs in breast cancer drug resistance^4^. Other work has demonstrated that *CEBPZ*, characterized as RBP and transcription factor (TF), is associated with maintaining the leukaemic state^8^. Yet another study has described the pro-tumorigenic role of the splicing factor *RBM39* in various cancers^9^. Furthermore, in liver cancer, prognosis-related RBPs such as *EEF1E1*, *LIN28B*, and *XPO5* have been identified^10^. Beyond these, *PES1* has been shown to influence patient survival and promote proliferation and tumorigenesis through the PI3K/AKT pathway^11^. Given that RBPs frequently co-bind their targets and participate in many processes, studying their interactions is of substantial interest^12^. However, the RBP regulome in cancer remains poorly investigated, making its exploration essential. This work focuses on uncovering the liver cancer regulome, as liver cancer is among the leading causes of cancer-related deaths and remains a major therapeutic challenge^13^. Previous studies investigated interactions among genes in liver cancer due to their importance in determining the treatment. For instance, activation of *EGFR* has been shown to reduce the effectiveness of the drug lenvatinib in liver cancer^14^. Additionally, *LIN28B-AS1* and *IGF2BP1* bind to each other and promote liver cancer progression in human cells^15^.

In recent years, gene regulatory networks (GRNs) have been successfully employed to analyze the mechanisms underlying various cancers^16–18^. For example, GRN inference allowed characterizing regulatory mechanisms of RBPs in pluripotency^19^. GRNs estimate and display interactions between regulators and their targets in biological systems using various omics data. In such graphic representations, nodes represent genes, and edges (or links) correspond to interactions between genes, the latter can be directed and signed. A common challenge in GRN research is the high level of noise in biological data, which increases the likelihood of inferring false positive (FP) interactions. Additionally, a study has described that the performance of GRN inference methods varies across different datasets^20^. Given such heterogeneity, devising an optimal solution to infer an accurate GRN is not a trivial task. One strategy is the consensus approach, inspired by the “wisdom of crowds” (WOC) principle where collective insights are stronger than an individual. Combining multiple GRNs allows for the construction of a more accurate consensus GRN^21^. This integrative approach leverages the strengths of different methods, reducing FPs and enhancing reliability. To this end, several consensus approaches have been developed on biological data to support disease characterization and treatment^22,23^. Moreover, several tools for the consensus GRN approach following the WOC idea have been created. For instance, a package designed for inferring consensus GRNs demonstrated increased robustness and performance in benchmarking studies^23^. Another example is GENECI, which also achieves high-quality GRN inference through a consensus-based approach^24^.

In this study, we aimed to uncover the liver cancer regulome and identify interactive RBPs as potential candidates for further research and therapeutic development. To achieve this, we employed a consensus GRN approach, integrating perturbation-based state-of-the-art regression and machine learning methods^25,26^. A recent benchmarking study emphasized that using knowledge about the perturbation is essential to infer an accurate GRN^27^. The consensus GRN was constructed from the short hairpin RNA knockdown followed by sequencing (shRNA-seq) data from the the encyclopedia of DNA elements (ENCODE), specifically generated for HepG2 cells, with a focus on a set of RBPs. To validate our methodology, we performed benchmarking of the consensus approach with synthetically generated shRNA-seq ENCODE-like data. The resulting liver cancer consensus GRN underwent comprehensive validation using both in-house and public RBP-RNA interaction data as main positive controls, including eCLIP-seq and RAP-seq^28^. Among multiple targets, *IGF2BP1* was a particular focus, as it is the most studied member of the IGF2BP family, regulating key mRNAs in tumorigenesis and stabilizing oncogenic transcripts. Its RNA-binding activity can be altered by cancer-associated mutations, most notably the recurrent R167C/H substitution^29–31^. Additionally, we identified modules in liver cancer GRNs and performed module-specific gene enrichment analysis. To further explore RBP-regulated pathways, we performed gene enrichment analysis on common targets of RBP pairs. Finally, we performed in silico drug repurposing analysis on the entire GRN as well as selected targets. Taken together, our multi-step, consensus approach enabled us to systematically characterize the key components of the liver cancer regulome, providing valuable insights for future research and therapeutic strategies.

## Results

### The liver cancer regulome highlights *AQR*–*PES1* interaction

**Fig. 1:**
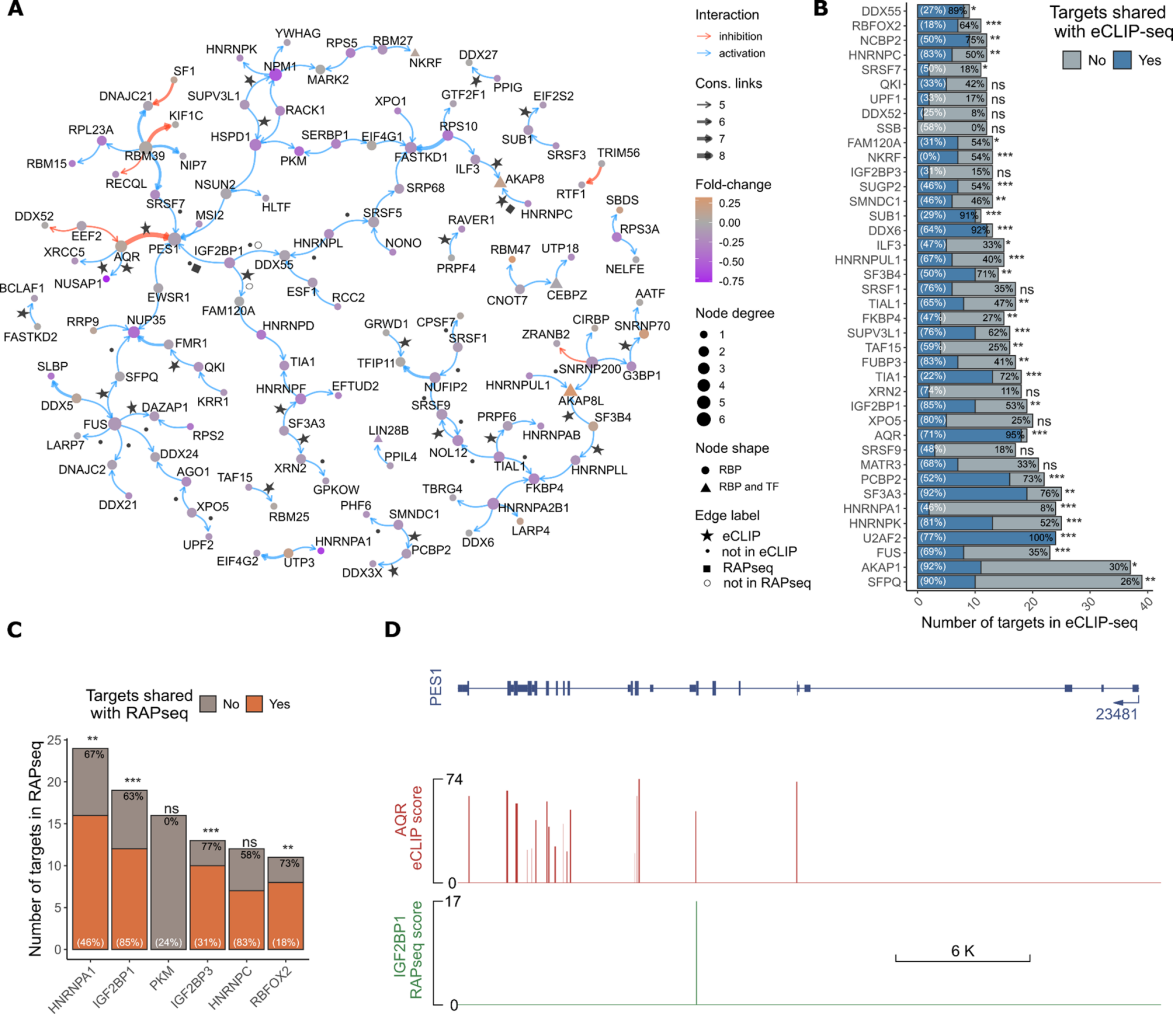
The regulome of liver cancer constructed as a consensus GRN. (**A**) Consensus GRN illustrates five minimum consensus links (5 +). Edge width indicates the number of GRNs supporting the edge; node color indicates expression Log_2_ fold-change in perturbed HepG2 cells over control; and edge label indicates significant (FDR < 0.05) binding in eCLIP-seq or RAP-seq data. (**B**, **C**) Bargraphs show topmost hubs (outgoing node degree > 10) from the 2 + consensus GRN validated with eCLIP-seq and RAP-seq data. Only RBPs that include eCLIP-seq or RAP-seq data are shown in the plot. For each gene, a hypergeometric test was performed (not significant (ns): *p* $\ge$ 0.1; *: *p* = [0.05, 0.1]; **: *p* = [0.01, 0.05]; ***:* p* $\le$ 0.01) to assess if a significant number of its targets are shared between the consensus GRN and significant (FDR < 0.05) experimental peaks. The shared links to targets are marked as the percentage of undirected RBP-RBP links in the GRN that overlap significant RBP-RBP interactions in eCLIP-seq or RAP-seq. For each gene, its percentage of outgoing links in the GRN is given in white font. (**D**) Genome tracks showing AQR eCLIP-seq and IGF2BP1 RAP-seq binding signals to *PES1* exonic (blue boxes) and intronic (blue line) regions. Only significant (FDR < 0.05) peaks are shown. The number indicates gene ID.

To infer a reliable GRN of RBPs in liver cancer cells, we developed a consensus approach (Supplementary methods, Figs. S1, S2, S3 and S4) and applied it to the ENCODE shRNA-seq knockdown dataset. The complete consensus GRNs for HepG2 and K562 cells are available in Supplementary Tables S1 and S2, respectively. Importantly, the results are focused on HepG2, while K562 was used as a pseudo-control for HepG2 GRN comparison. Notably, three interactions *AQR*-*PES1*, *RBM39*-*KIF1C*, and *FASTKD1*-*RPS10* were identified by seven or more methods, suggesting a high likelihood of being true positive links. Focusing on the liver cancer regulome, we analyzed the HepG2 5 + consensus GRN, i.e. supported by five or more GRNs, which consists of 117 regulatory interactions (Fig. 1A). Interactions supported by five or more methods (5 + threshold) were prioritized due to their high benchmark precision (0.70), suggesting a strong enrichment for true positive links (Figs. S2 and S3). This threshold also ensures relatively high network coverage and readable density, with an average outdegree of 1.65. In addition, many regulatory interactions in the HepG2 5 + consensus GRN are supported by significant RBP–RNA binding events detected in eCLIP-seq and RAP-seq datasets, providing semi-quantitative experimental evidence consistent with the predicted regulatory links (Fig. 1A).

To further characterize regulatory RBPs, we employed data from eCLIP-seq and RAP-seq. For RBP hubs identified in the undirected + 2 GRN, we evaluated whether their predicted RBP targets were significantly enriched among experimentally detected targets. This was assessed quantitatively using a hypergeometric test, which determines whether the observed overlap between predicted and experimentally supported targets is greater than expected by chance. (Fig. 1B and C). Here, we focused on the 2 + GRN as a denser network, increasing the likelihood of validating less supported links using positive control binding data. This analysis highlighted the significant roles (*P* < 0.05) of *U2AF2* and *AQR* as major RBP regulators as well as *SUB1* and *DDX6* as minor RBP regulators in liver cancer, with over 90% of their targets supported by significant false discovery rate (FDR) < 0.05 eCLIP-seq binding events (Fig. 1B).

Additionally, we demonstrate that the interaction of *AQR*-*PES1*, inferred by eight methods, showed significant (FDR < 0.05) eCLIP-seq binding (Fig. 1D). Furthermore, we utilized RAP-seq data to validate binding of IGF2BP1, an RBP previously linked to liver cancer, and identified *PES1* as one of its target genes (inferred by five methods). In the literature, *AQR* was described as an RBP that encodes an RNA helicase and contributes to the accumulation of DNA damage upon knockdown in colon cancer cells^32^. This shared regulatory interaction points to a possible functional overlap between *PES1* and *IGF2BP1* in liver cancer cells. Moreover, *PES1* has been found to be overexpressed in liver cancer and influence the patient’s survival^33^. Overall, these results indicate hypothetical regulatory interactions among RBPs in liver cancer and propose possible involvement of key RBPs, including *AQR*, *PES1*, and *IGF2BP1*, in cancer-associated processes.

### Novel RBP interactions and survival associations identified from validated GRNs

**Fig. 2:**
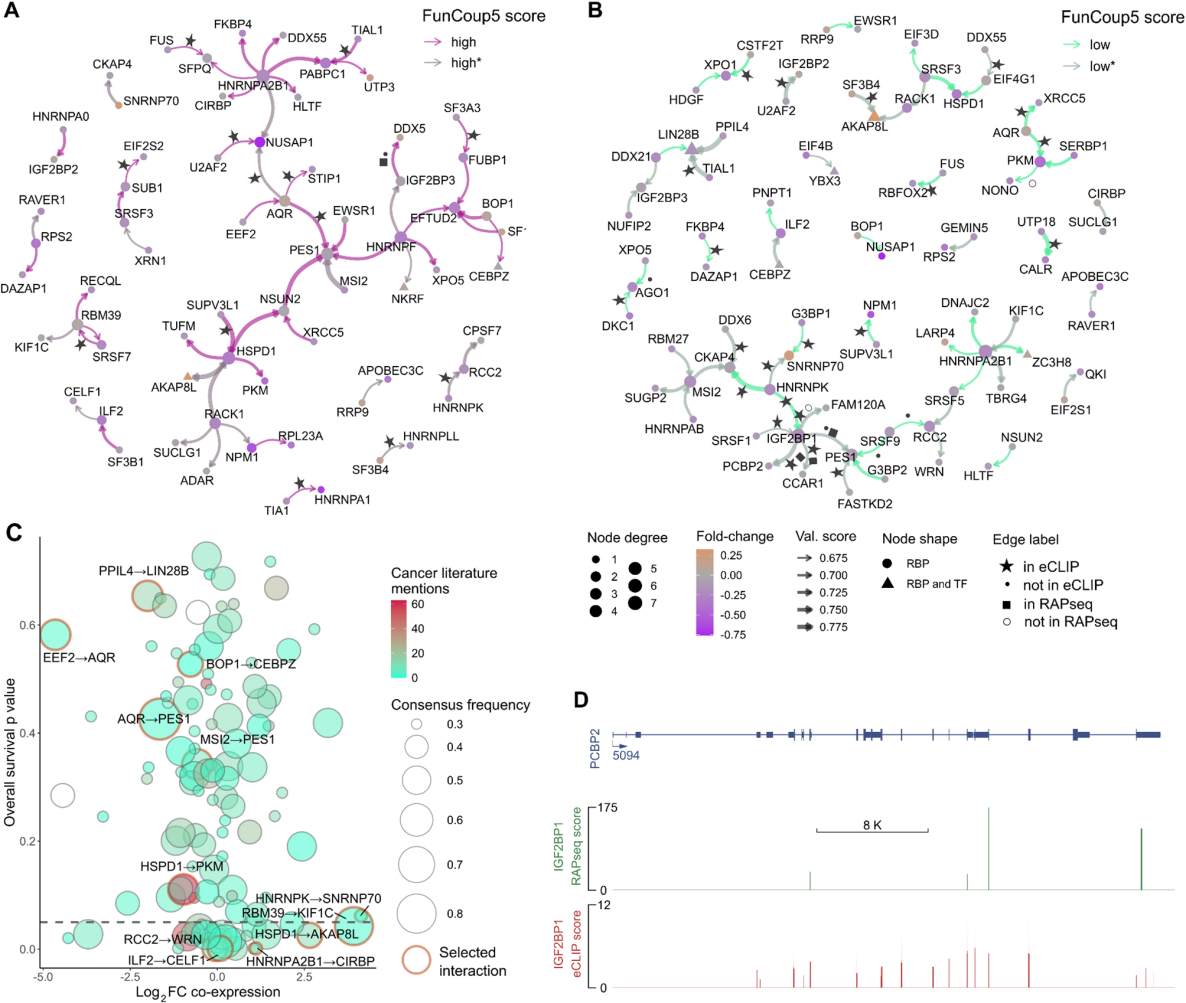
GRN and its validation for a set of RBPs related to liver cancer by DisGeNET. (**A**) GRN of RBPs with high FunCoup score and related to liver cancer. Edge width indicates the number of GRNs supporting the edge and edge colors indicate a high FunCoup score (bright violet labeled as high) and predicted high FunCoup score (gray-violet labeled as high*). (**B**) GRN of liver RBPs with low FunCoup confidence score and related to liver cancer. Edge width indicates the number of GRNs supporting the edge and edge colors indicate a low FunCoup score (bright seagreen labeled as low) and predicted low FunCoup score (gray-seagreen labeled as low*). (**C**) Validation of the liver cancer regulome using the effect of RBP expression on patient overall survival (harmonic mean *P* value), co-expression fold-change (FC), and RBP cancer literature popularity. Interaction pairs are shown with RBP names for some of the noteworthy predicted interactions listed in Supplementary Table S5 and Fig.S13. The dashed line indicates a *P* value of 0.05. (**D**) Genome track exemplifies IGF2BP1 eCLIP-seq and RAP-seq binding signals to *PCBP2* exonic (blue boxes) and intronic (blue line) region. Only significant (FDR < 0.05) peaks are shown. The number indicates gene ID. The signed GRN of (**A**) and (**B**) is available as Fig. S6A.

To characterize the regulome of RBPs related to liver cancer, we performed an iterative enrichment analysis using DisGeNET, as detailed in the Methods section and Fig. S5. Here, we selected the 3 + GRN as its average node degree best reflected the density of real GRNs (Fig. S1B). We constructed the liver cancer related RBP regulome (Fig. 2) by analyzing multiple hypergeometric tests across all liver cancer gene set enrichments (FDR < 0.05). The local maximum of -log_10_(*P*) was then identified, resulting in a subset of 119 top links (Fig. S5B). In addition, we divided the GRN into two subGRNs corresponding to high and low FunCoup (v5) scores, reflecting either high or low FunCoup confidence scores of functional associations (Fig. 2A and B). The FunCoup evidence scores were discretized into low and high using equal frequency binning cutoffs. Both subGRNs can indicate pathway activation or complex formation in response to changes in a cell. Notably, in the low-scored subGRN, *IGF2BP1* emerged as a master regulator of other RBPs, confirmed with significant binding (FDR < 0.05) in eCLIP-seq and RAP-seq experiments (see Fig. 2B). Moreover, we also detected interactions absent in the FunCoup database, suggesting that novel elements in the liver cancer regulome were found. A complete liver cancer-related GRN (subset of 3 + GRN) is available in Supplementary Materials (Fig. S6A). The information about applied databases can be found in Supplementary Table S4.

*IGF2BP1* is a notable RBP relevant to cancer pathways as previously investigated^34^. Given the availability of both eCLIP-seq and RAP-seq data for *IGF2BP1*, we merged its significant targets from both experiments and performed enrichment analysis on such a set (Fig. S6B-D). Our analysis revealed that *IGF2BP1* targets were associated with various disease states, including cancer and immune response. Its targets are also significantly related to Rho GTPases and the activation of the MYC pathway, as well as various transport and localization biological processes. Notably, the MYC signaling pathway is regulated by one of the RBPs, i.e. *NELFE*, in liver cancer^35^. Another study showed that oncogenesis in lung cancer is related to the regulation of the MYC pathway via *HNRNPK*^36^. These two examples highlight the regulatory role of RBPs in the MYC pathway.

In the Supplementary Material, we provide a validation summary of all 119 liver cancer-related interactions (Fig. S7) and a full comprehensive validation list of 648 interactions (full 3 + GRN) (Supplementary Table S3). Predicted regulatory interactions were evaluated using quantitative integrated confidence scoring, in which multiple supporting features, including eCLIP-seq and RAP-seq information, were scaled between 0 and 1 and combined into a single score to rank interactions. Selected interactions were further investigated (Fig. 2C). For example, *HSPD1-AKAP8L* and *ILF2-CELF1* involve RBPs that strongly affect the survival of liver cancer patients, despite being understudied on cancer-related RBPs. We also observed a group of interactions that do not significantly affect survival (points above the dashed line in Fig. 2C). Another example is *RBM39*-*KIF1C,* which exhibited high co-expression fold-changes and impact on survival but has low literature popularity. Conversely, *HSPD1*-*PKM* included RBPs that were highly mentioned in the literature concerning cancer. The oncogenic role of *HSPD1* has been investigated in oral and breast cancers^37,38^, while oncogenic activities of *PKM* were confirmed in thyroid and colorectal cancer^39,40^. Furthermore, we investigated combined expression-related effects on survival by merging discretized gene expression into low-low, low–high and high-high corresponding to each gene–gene interaction in the full 3 + GRN (Fig. S8). This revealed that a large number of connected genes in this GRN together have a significant impact on survival, and that *SFPQ* interacts with many other RBPs that together affect survival. In summary, multi-layer validation with the resources listed in Table 2 highlights the recovery of known interactions and proposes novel potential interactions between RBPs in liver cancer.

### Validation of TFs reveals a hypothetical link between *CEBPZ* and *RBM27*

**Fig. 3:**
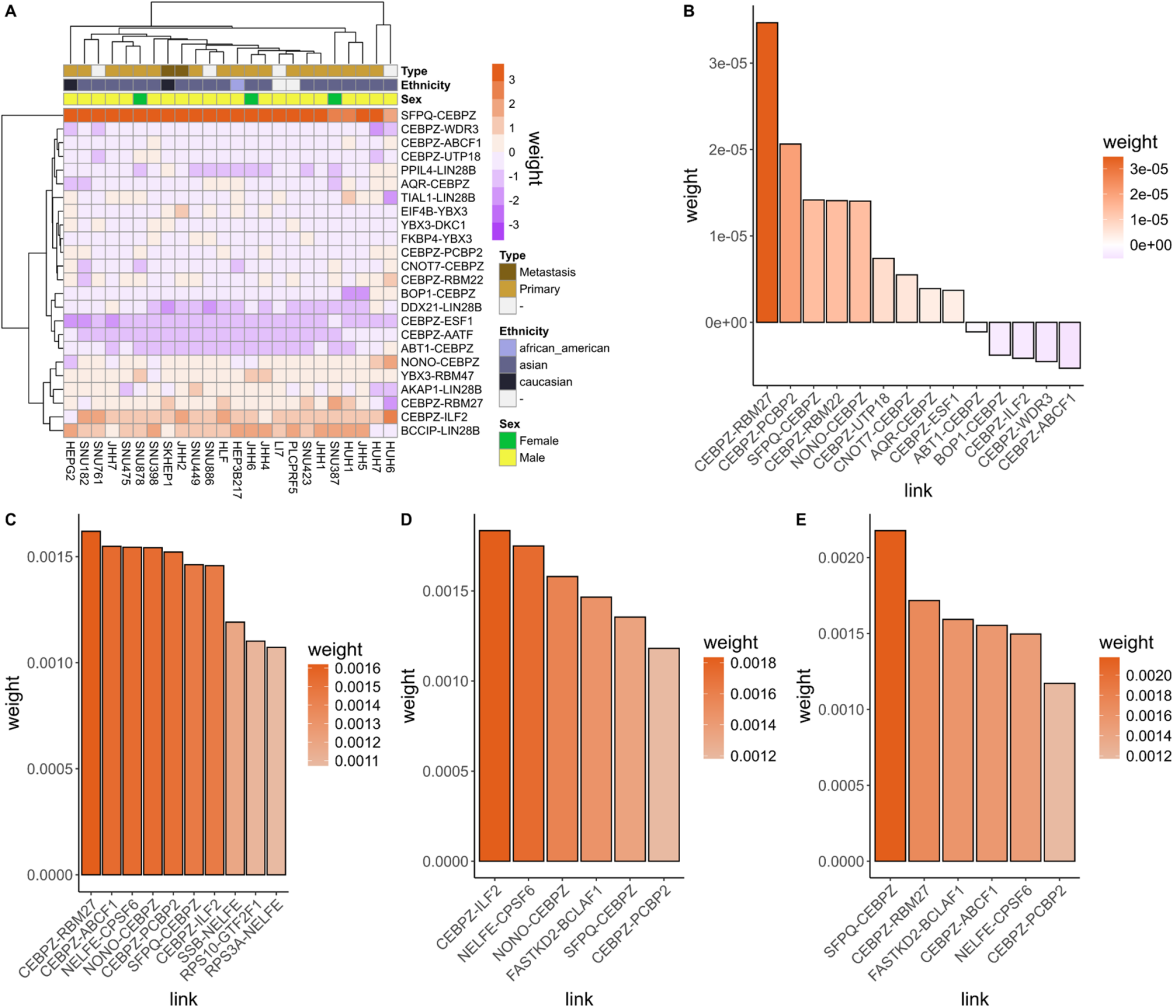
Validation of 24 interactions in the 3 + GRN with the GRAND and GRNdb databases. Weights on the plots represent the values given by certain inference methods as an indicator of links. (**A**) intersection with 24 GRNs from liver cancer cell lines inferred with LIONESS. In addition, clinical data was marked on the heatmap. (**B**) intersection with a GRN from TCGA-LIHC performed with OTTER (**C**) Intersection with GRNdb liver tumor regulons single-cell data generated with GENIE3. Weight is GENIE3 importance. (**D**) Intersection with GRNdb healthy liver tissue regulomes single-cell data generated with GENIE3. Weight is GENIE3 importance. and (**E**) Intersection with GRNdb liver peripheral blood regulomes single-cell data generated with GENIE3. Weight is GENIE3 importance.

Among the analyzed RBPs, several proteins are also annotated as TFs based on GRAND and GRNdb. Because these proteins can function both as canonical DNA-binding TFs and as RNA-binding proteins, we sought to contextualize the regulatory relationships inferred from our knockdown RNA-seq–derived GRNs. To this end, we compared the interactions identified in our 3 + consensus GRN with publicly available GRNs from GRAND and GRNdb, which provide precomputed interactions for liver cancer and healthy liver tissues based on large transcriptomic datasets. We intersected all edges from our consensus GRN with those reported in these resources and compiled an undirected network to explore potential regulatory relationships common across datasets. Notably, this comparison does not distinguish between transcriptional and post-transcriptional mechanisms but provides independent support for the inferred associations. All intersections are shown in Fig. 3. In the following section, we highlight several highly ranked TF–RBP interactions selected based on their high importance scores in our network and their absence in healthy liver tissue.

The analysis revealed a strong regulatory interaction between *SFPQ* and *CEBPZ* across all 24 liver cancer cell lines (Fig. 3). However, it is also reflected in the healthy liver GRNs (Fig. 3). To investigate whether this regulation could stem from TF-DNA binding interactions, we queried ChIP-seq data for *SFPQ* in the Cistrome database (v3.0)^41^, but no binding to *CEBPZ* was found in the four included HepG2 cell lines, indicating that what we observe stems from TF RBP activity.

Another prominent interaction is *CEBPZ*-*RBM27*, which was absent in a healthy liver, but present in all investigated GRNs of liver cancer cells. Overall, CEBPZ is predicted to be associated with multiple RBPs, such as RBM22, ESF1, and ABCF1, highlighting potential regulatory interactions that require experimental validation. It has been shown that *CEBPZ*, also known as *CTF2*, physically interacts with *TP53*^42^. *RBM27* is not frequently mentioned in the context of cancer regulation, but its role in RNA decay has been discovered^43^. These findings highlight the potential involvement of TFs like CEBPZ in modulating the expression of RBP genes in a cancer cell-specific context.

### Module detection identifies groups of RBPs linked to cancer pathways and MYC targets

To perform a module enrichment analysis, we utilized two GRNs, namely the 5 + consensus GRN (Fig. 1A) and the 3 + consensus liver cancer-related GRNs (Figs. 2A, B and S6A) to investigate two various scenarios. These include interactions: (1) related to liver cancer (top 119 from 3 + GRN) and (2) highly precise based on the benchmark (5 + GRN). We observed that several modules contained RBPs related to the oncogenic transcription factor *MYC* (Table 1). Previous reports established that *MYC* plays a crucial role in liver cancer initiation and progression^44^. Notably, the light green module (Fig. 3A) includes four out of six RBPs (*NPM1, RACK1, HSPD1* and *SUPV3L1*) that were identified as *MYC* targets. This suggests a hypothesis that the remaining two RBPs, *YWHAG* and *HNRNPK,* could be novel *MYC* targets. Members of the YWHA and HNRNP families have previously been identified as *MYC* targets^45,46^. Within the same module, two RBP *MYC* targets from the MsigDB gene set HALLMARK_MYC_TARGETS_V2, *NPM1* activation by *SUPV3L1*, are connected. Their interaction was confirmed by eCLIP-seq, suggesting liver cancer-relevant regulation occurring in the *MYC* pathway. To validate these, we investigated co-expression mechanisms between *MYC* and the potentially novel *MYC* targets (Fig. S9). For instance, strongly significant dependence (*P* < 0.001) between *MYC* and *PKM* in healthy tissue that is absent in liver cancer (Fig. S9J).

**Table 1:** Hallmark *MYC* targets and connected RBPs found in GRN modules. Novel *MYC*-related genes are underlined.

RBPs related to hallmark MYC targets V1 and V2 in GRN modules (Fig. 4)	RBPs shared with MsigDB HALLMARK_MYC_TARGETS_V1	RBPs shared with MsigDB HALLMARK_MYC_TARGETS_V2
*ADAR, AKAP8L, CCAR1, EIF2S2, EIF3D, EIF4G1, FAM120A, FASTKD1, HNRNPK, HNRNPLL, HSPD1, IGF2BP1, NPM1, PCBP2, PKM, RACK1, RPL23A, SERBP1, SF3B4, SRSF1, SRSF3, SUB1, SUPV3L1, TUFM, XPO1, XRN1, YWHAG*	*EIF2S2, EIF3D, FAM120A, HSPD1, NPM1, RACK1, SERBP1, SRSF1, SRSF3, TUFM, XPO1*	*HSPD1, NPM1, SUPV3L1*

**Fig. 4:**
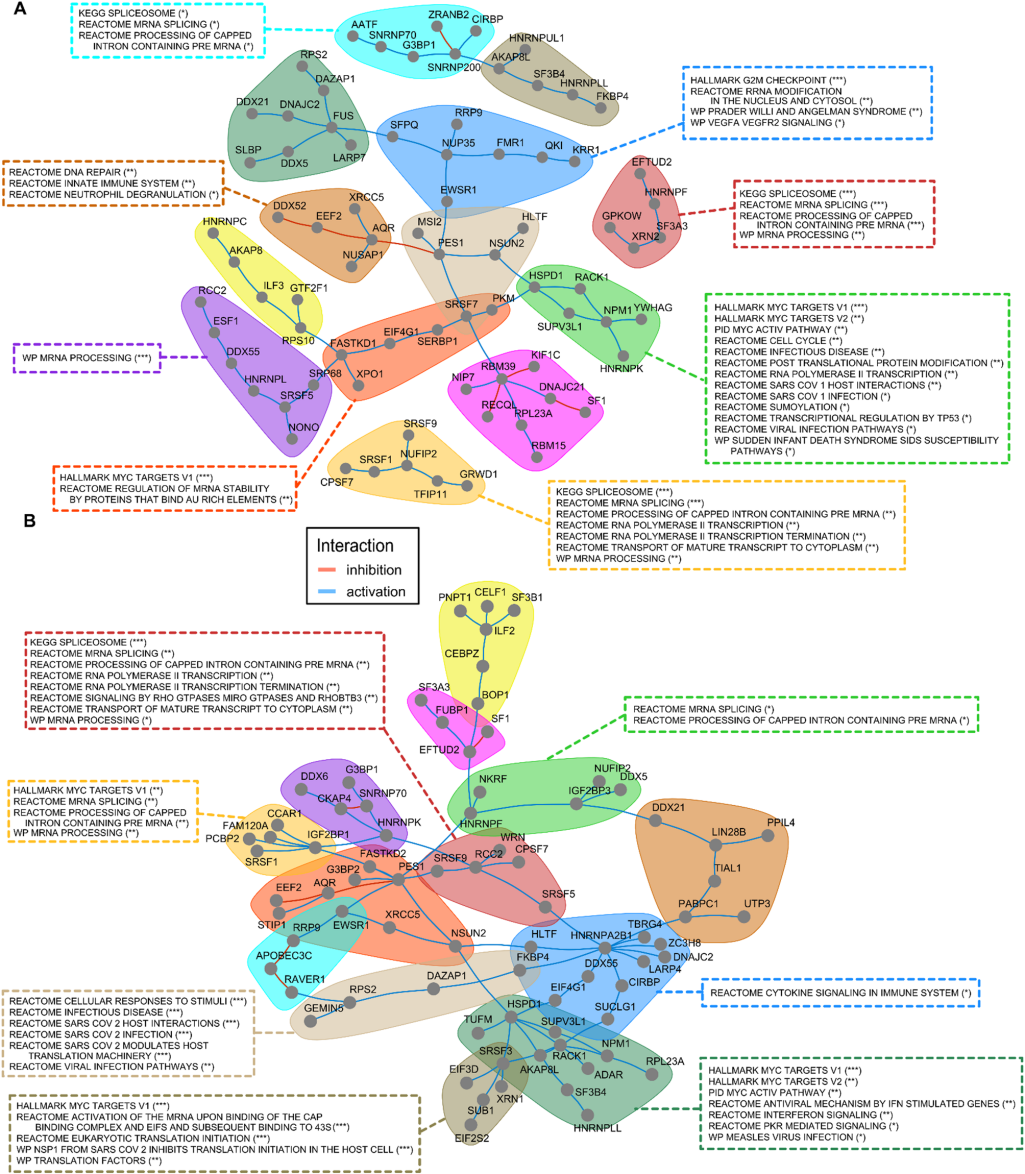
Module detection and MSigDB (C2, C5, C6, and H) enrichment of modules. Selected and simplified significant (FDR < 0.05) terms are included in the legend. As a background, a full list of RBPs was taken. Some terms were merged and simplified according to their similarity. (**A**) The consensus GRN where at least 5 methods support each link. Modules are color-coded and pathway enrichment terms are displayed. (**B**) GRN of 119 liver cancer-related links. Terms include FDR-corrected* p*-values as follows *p* $\ge$ 0.1; *: *p* = [0.05, 0.1]; **: *p* = [0.01, 0.05]; ***:* p* $\le$ 0.01.

Moreover, we identified clusters related to various other cancer pathways. These included VEGFA/VEGFR2, Rho GTPases and DNA repair. For instance, the *AQR*-*XRCC5* interaction included in the light brown module (Fig. 4A) contained RBPs involved in DNA repair pathways. Another noteworthy module is that of *IGF2BP1* (Fig. 4B, light orange module), which was enriched with RBPs involved in mRNA splicing and processing of capped intron among other pathways^47^.

Several modules were also enriched with splicing-related terms, indicating the crucial role of splicing performed by RBPs during oncogenesis^48^. We found distinct splicing modules (and in Fig. 4A dark red module and Fig. 4B light orange clusters). Another significant enrichment was found for the immune system and infectious disease pathways in multiple clusters. This finding aligns with the growing recognition that immunotherapy is a promising strategy for treating liver cancer patients and improving their survival^49^.

Overall, our results identified pathways affected in liver cancer cells upon RBP knockdown and revealed potentially novel *MYC* targets that warrant further investigation for their carcinogenic potential.

### Shared targets of RBP pairs highlight activation of cancer pathways

**Fig. 5:**
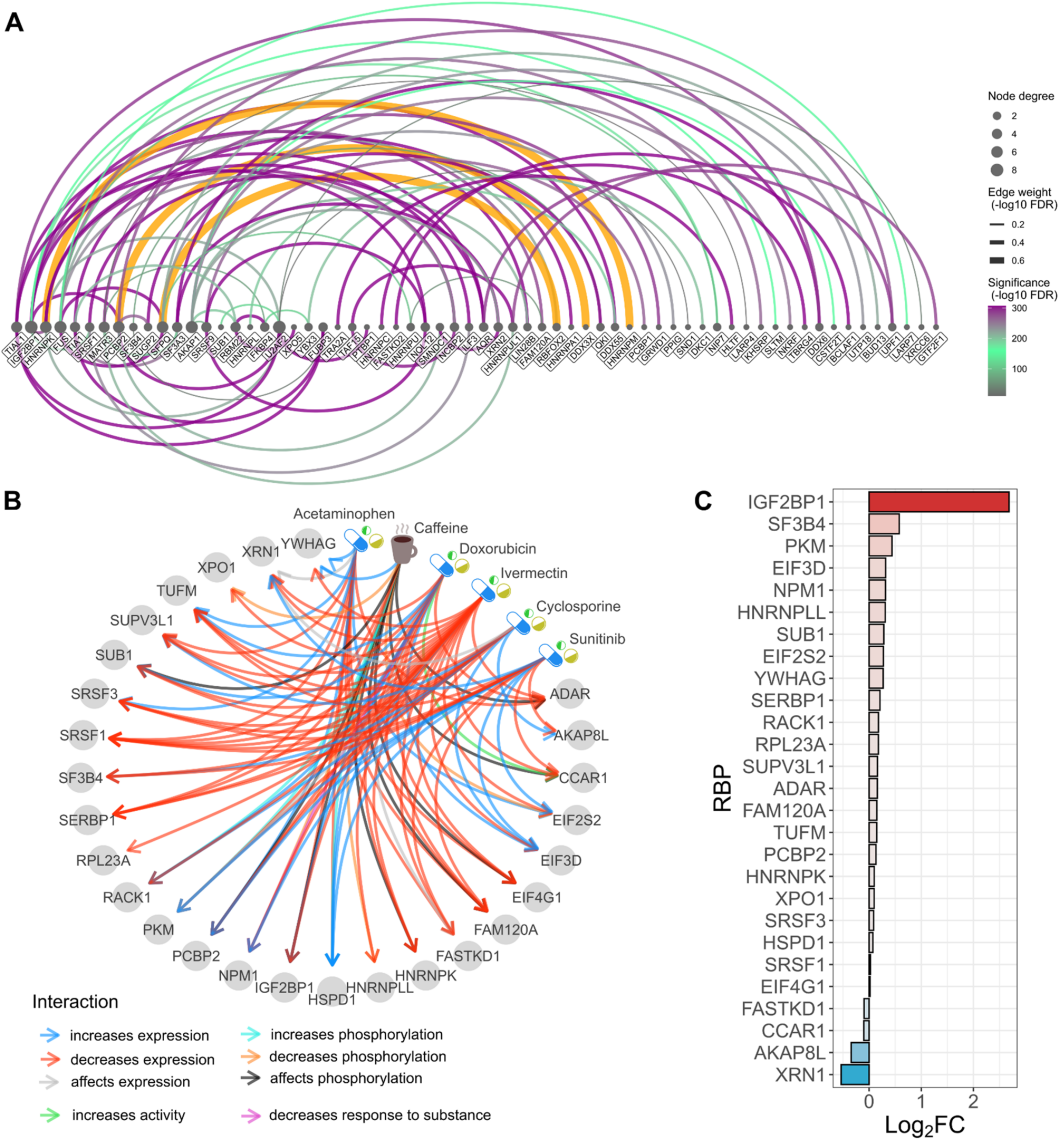
(**A**) Map of targets shared (only significant overlap FDR < 0.05) between RBP pairs in eCLIP-seq and RAPseq data based on the 3 + GRN estimated with Fisher’s exact test. The thickness of the arch indicates a -log10* P* value. The size of a node indicates the number of connections in the diagram. (**B**) Drug repurposing and patient-based expression of *MYC*-related RBP targets (Table 1). CTD-derived interactions between drugs and RBPs. The color of the edges represents various types of interactions. Drugs are marked as nodes with special icons. (**C**) Log_2_ fold-change (Log_2_FC) based on expression from TCGA and GTEx cohorts. The colors of the bars represent the negative (blue) or positive (red) Log_2_FC. Values were calculated as median RBP expression in LIHC over median RBP expression in healthy liver.

We next examined the complete regulome of the 3 + GRN. We investigated all interacting RBP-RBP pairs to search for pairs with overlapping targets, using all RNA targets (beyond the set of RBPs) derived from eCLIP-seq and RAP-seq experiments (Fig. 5A and S10). We interpret that a high number of common targets between any two RBPs suggests a shared regulatory circuitry, possibly through co-dependency in their mode of action or a direct interaction between both partners to bind their RNA targets. For this, we employed Fisher’s exact test. As a result, we discovered that three RBP-RBP interacting pairs: *PCBP2*-*DDX3X*, *HNRNPK*-*HNRNPM* and *SFPQ*-*RBFOX2*, share the most significant number of the protein coding targets. Members of the heterogeneous nuclear ribonucleoprotein (hnRNP) family have been described as the main regulators of alternative splicing^50^. We found that the *HNRNPK*–*HNRNPM* interaction scored highly in the FunCoup network, with a confidence level of 1. Enrichment analysis of their common protein coding targets indicated potential involvement in various cancer pathways, such as the Rho GTPases cycle, platelet-derived growth factors (PDGF), or VEGFA/VEGFR2 signaling (Fig. S10C). *HNRNPM* has been identified in the literature as a candidate therapeutic target in liver cancer^51^. The *SFPQ*-*RBFOX2* interaction showed enrichment patterns similar to *HNRNPK*-*HNRNPM*, but also included the EGF/EGFR signaling pathway (Fig. S10D). Both *SFPQ* and *RBFOX2* belong to a group of splicing factors^52^. Lastly, the *PCBP2*–*DDX3X* interaction is associated with diseases related to growth factor–mediated signal transduction, in addition to several cancer-related pathways such as TP53 and VEGFA/VEGFR2 (Fig. S10B). *DDX3X* and *PCBP2* modulate innate immunity by controlling cell stress responses and inflammasome activation, or by regulating antiviral signaling through mitochondrial antiviral-signaling protein degradation, respectively^53,54^. These findings underscore the complex regulatory interactions involving RBPs and their theoretical roles in cancer and immune response pathways.

### RBP-targeting drug candidates identified via in silico GRN-Based repurposing

To conduct in silico drug repurposing on the liver cancer regulome, we carried out several analyses. First, we investigated the entire consensus 5 + GRN in CLUEreg, which revealed Irinotecan as the topmost drug based on cosine similarity. This suggested that HepG2 cells undergoing RBP knockdown may exhibit similar mechanisms to those induced by Irinotecan (Table S6), a well-known anticancer drug used for colorectal cancer treatment as it is an inhibitor of DNA topoisomerase I^55^.

Second, by reapplying CLUEreg, we evaluated *IGF2BP1,* consistent with a previous study that investigated its binding sites in HepG2^56^. We analyzed common eCLIP-seq and RAP-seq-based targets of *IGF2BP1* (Fig. 2, Fig. S6B-D). Our analysis identified Garcinol or Gallic-acid as potential agents to reverse the expression profile of IGF2BP1. Garcinol inhibits STAT3, and Gallic-acid arrest cells at the G2/M phase. STAT3 has been shown to play a crucial role in cancer inflammation and immunity, aligning with the results of our enrichment analysis (Fig. S6C-D). In addition, we investigated the differentially expressed RNA targets of *AQR* and *U2AF2* detected in the 2 + GRN (Fig. 1B). Notably, our analysis suggests that the effects of targets of the two major regulators *AQR* and *U2AF2* could potentially be modulated by WNT-related drug treatments (Table S6)^57,58^.

Finally, based on CTD, we found several chemotherapeutic drug candidates, such as Doxorubicin and Ivermectin (Fig. 5B-C, Fig. S11). Recent studies have highlighted the role of these drugs in liver cancer therapy^59,60^. Our analysis implies that several drugs contribute to inhibiting or activating RBP *MYC* targets. For example, Ivermectin and Acetaminophen decrease the expression of *IGF2BP1*^60,61^, which is up-regulated in patients (Fig. 4C). Moreover, both Doxorubicin and Ivermectin decrease the expression of *PES1,* which is elevated in liver cancer patients (Fig. S11). In addition, we identified phosphorylation-related interactions between caffeine and MYC-associated targets. Multiple studies have proposed the hypothesis that coffee consumption may reduce the risk of various cancers, including liver cancer^62,63^.

In sum, our findings highlight several promising drug candidates and potential therapeutic targets for liver cancer, providing options for treatment strategies based on modulating RBPs and their regulatory network. However, these hypotheses should be further confirmed experimentally.

## Discussion

Mechanisms behind cancerogenesis are complex and often involve interactions among molecules that reflect the activation or deactivation of certain pathways. Multiple studies attempted to estimate regulatory networks for liver cancer, yet not focused on RBPs and often utilized only a single inference method^64–66^. The RBP regulome of liver cancer represents crucial processes that may lead to activations and inhibitions in human cancer cells. To design treatment it is valuable to recognize these processes and provide hypotheses of binding mechanisms that influence the expression of RBPs and their targets. Furthermore, it is important to handle noise in gene expression data that can lead to FP interactions. To produce a regulome in a more accurate and interpretable way, a consensus GRN approach can be applied. To this end, we utilized 10 various GRN inference methods to reveal the liver cancer regulome by following the WOC approach^21^ and further performed a comprehensive analysis.

A substantial outcome of our work is a set of computationally-derived interactions among 232 RBPs in liver cancer. Based on benchmarking with the ENCODE-like data, we believe that several RBP-RBP regulations are very likely to be true positives, namely *AQR*-*PES1*, *RBM39*-*KIF1C,* and *FASTKD1*-*RPS10*. Notably, *AQR*-*PES1* was detected by 8 out of 10 methods, and the eCLIP-seq experiment further confirmed the binding of these two RBPs. Co-expression of *AQR* and *PES1* is lower in liver cancer than in healthy control (Fig. S12), suggesting that their functional association may be reduced in liver cancer cells.

Another finding is the set of *MYC*-related RBP targets detected based on the module enrichment analysis of GRNs. As RBPs share many common mechanisms related to post-transcriptional processes, performing an enrichment analysis presented a considerable challenge. Thus, we used a full list of known RBPs as a background. The enrichment results agree with the previous work^67^ and show the important role of RBPs in the MYC pathway. We also observed that some clusters (Fig. 4) corresponded to other cancer-related pathways, e.g. DNA repair, immune system response and splicing^68,69^. In our in silico drug repurposing analysis, we identified several candidate compounds previously reported to target cancer-related pathways. The predicted drug–target interactions suggest potential effects on apoptosis and other major oncogenic processes. For instance, sunitinib is an inhibitor of *SF3B4* and an activator of *XRN1* that are upregulated and downregulated in liver cancer, respectively (Fig. 5). Sunitinib is an approved tyrosine kinase receptor inhibitor used clinically for cancer therapy^70^. As these findings are based solely on computational predictions, they represent preliminary insights that require experimental validation before any therapeutic implications can be drawn.

As a result of simulations, we share two synthetic ENCODE-like data, together with corresponding GRNs, so that they can be reused in future studies for benchmarking these cohorts (see Data Availability). We also share full consensus GRNs and a 3 + consensus validation table that can be a resource for future comparison of regulomes (Table S1-S3). The K562 cell line was also used in this work, however not analyzed as extensively as HepG2. This data and its consensus GRN are attached in supplementary materials to this work (Table S2). We believe that it can be comprehensively utilized in the future as a resource for interactions for studies on leukemia.

In this work, we aimed to create a consensus approach using only a perturbation-based method, thus the commonly applied GENIE3 was not considered. Being aware of the good performance of GENIE3 in benchmarks and other analyses, we used CART in the consensus approach. Furthermore, GENIE3 and its successor GRNBoost2^71^ consider all unperturbed genes in data, while here we focus only on perturbed, i.e. knocked-down, RBPs that allow us to compute a consensus GRN with better performance. As our analysis was restricted to GeneSPIDER-embedded methods and single-omics data from the ENCODE Project Consortium, the inferred networks likely capture only a subset of the underlying regulatory relationships. Future studies could extend this framework by incorporating a broader collection of GRN inference methods and integrating multi-omics datasets to construct a more robust consensus network. In particular, approaches that explicitly incorporate prior knowledge of TF–target interactions may further improve network accuracy. Such integration could increase the number of true positive regulatory interactions while preserving a sufficiently dense consensus GRN.

Importantly, this study is focused primarily on the transcriptome, leveraging multi-layer datasets, including shRNA-seq, RNA-seq, eCLIP-seq, and RAP-seq. By combining these complementary approaches, we capture multiple layers of post-transcriptional regulation while maintaining a focus on transcriptomic regulation. Consequently, a notable constraint of this study arises from the reliance on transcriptomics datasets and publicly available resources, which are subject to experimental and technical variability, including potential batch effects. To partially address this limitation, we further validated a subset of inferred interactions using the FunCoup functional association network, which integrates multi-omics evidence across species including transcriptomics, proteomics, genomics, regulomics, and comparative genomics. Furthermore, because the analysis relies on bulk liver cancer datasets, it may not fully capture the molecular and cellular heterogeneity specific to tumors. In addition, as this study was primarily conducted using HepG2 cells, i.e. hepatoblastoma-derived cells^72^, the findings may not fully capture the heterogeneity of other liver cancer subtypes or primary tumor tissues. Future studies integrating single-cell and multi-omics datasets could refine the GRNs and provide deeper insights into tumor heterogeneity across diverse hepatocellular carcinoma contexts.

As this study was primarily conducted using HepG2 cells, the findings may not fully capture the heterogeneity of other hepatocellular carcinoma subtypes or primary tumor tissues.

The perturbation data were limited to RNA-binding proteins (RBPs), and therefore other potential regulatory targets, such as protein-coding genes outside this group, were not included in the GRN inference. This restriction to 232 RBPs arises because the GRN inference methods we employed require perturbation data for all genes included. To the best of our knowledge, larger collections, having matched eCLIP-seq experiments, are not currently available. Nevertheless, this work deliberately focused on interactions among RBPs because of their central role in post-transcriptional regulation and cellular homeostasis (see Introduction). To partially address this limitation, we examined the downstream RNA targets of RBPs using data from eCLIP-seq and RAP-seq. Enrichment analysis of these experimentally supported targets (Fig. 3 and S6) allowed us to identify biological pathways associated with protein-coding genes regulated by RBPs.

While these complementary analyses provide functional insights, the majority of inferred regulatory interactions remain indirectly validated. Moreover, not all 232 RBPs analyzed here have been experimentally validated across both eCLIP-seq and RAP-seq datasets. Future targeted RBP–RNA binding experiments could corroborate predicted interactions, particularly for *RBM39*, which showed a regulatory role in our analysis but lacks corresponding ENCODE eCLIP-seq data.

In conclusion, our analysis outlines the RBP-associated regulome in liver cancer and proposes functional modules and drug candidates for future study. To the best of our knowledge, it is the first study that infers a liver cancer regulome of RBPs via a consensus approach using perturbation-based methods. The discovery of the liver cancer regulome relies on comprehensive benchmarking that reflects the high precision of GRNs using a consensus approach. Our findings brought known and novel interactions among RBPs. For the inferred consensus GRN, we performed comprehensive validation using external data sets and GRNs. Furthermore, we executed a GRN-based module enrichment analysis that revealed a set of known and potentially novel *MYC* targets. Our analysis detected several RBP-RBP interactions likely to participate in cancer-related pathways in the liver tissue. For instance, *AQR*-*PES1* is the strongest evidence in this analysis. Finally, we performed in silico drug repurposing that revealed a list of potential cancer drug candidates. Overall, the results from module enrichment analysis and drug repurposing can support the development of treatments for liver cancer patients.

## Methods

**Fig. 6:**
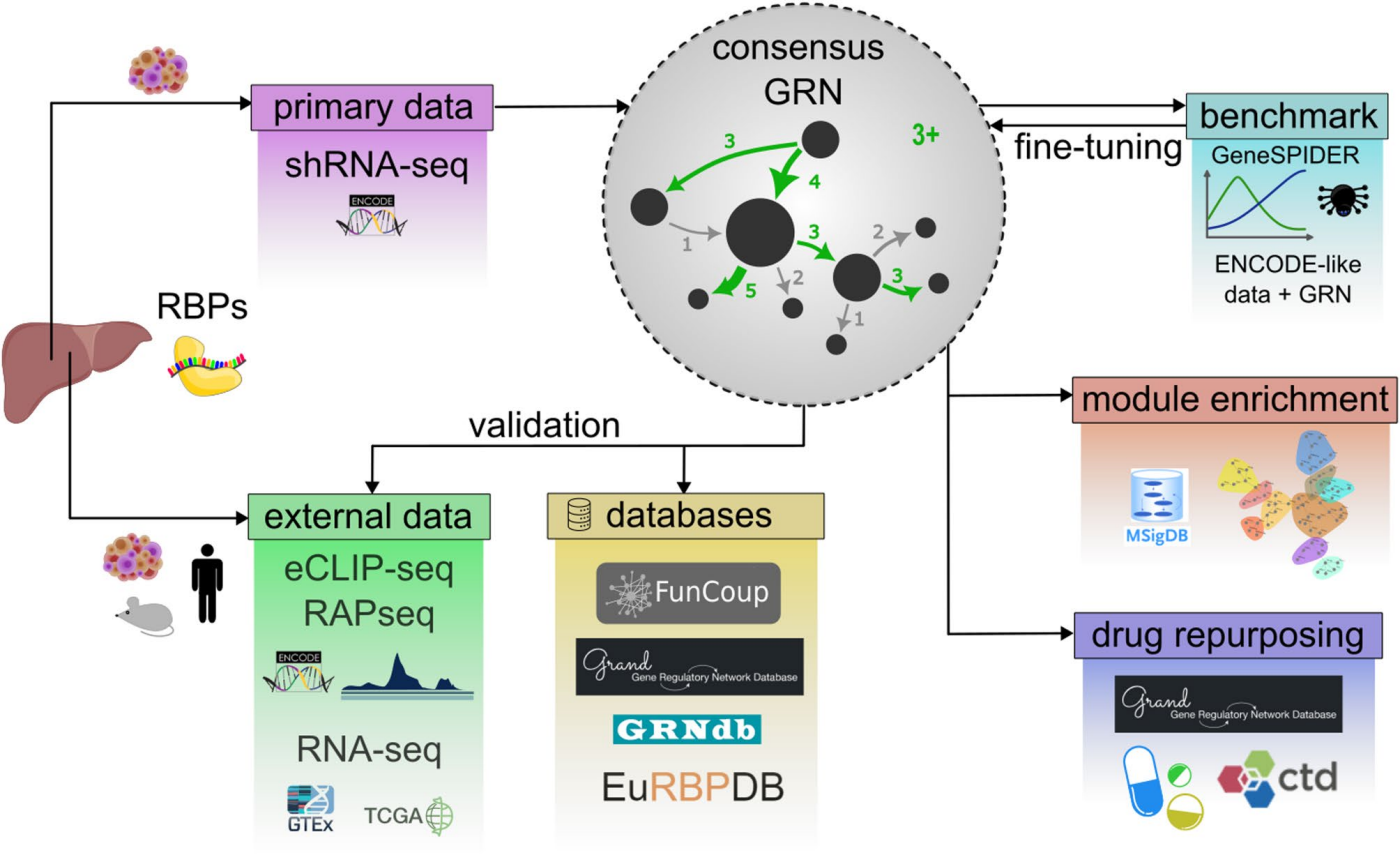
An overview of the analysis aiming at elucidating liver cancer mechanisms between RBPs.

This section includes a description of the data and methods used for the analysis. We also include a summary picture of our research (see Fig. 6).

### shRNA knockdown followed by RNA-seq

The GRN inference was performed using publicly available shRNA RNA-seq of the liver cancer cell line (HepG2)^73^, provided by the Encyclopedia of DNA Elements (ENCODE) project^74^. The ENCODE repository aims to generate and share data on the functional elements of the human genome. Additionally, we incorporated shRNA RNA-seq from the chronic myelogenous leukemia cell line (K562) to generate a corresponding GRN for comparing the interactions in HepG2, i.e. a pseudo-control GRN (pcGRN). Such a pcGRN was employed further as a reference GRN for discovering interactions specific to liver cancer (not present in the GRN of K562). In both ENCODE datasets, cells were treated with shRNA targeting 232 RBPs. To infer GRNs from these datasets, replicates were separated within samples to maintain the perturbation design required for the GRN inference methodology^26^. HepG2 and K562 datasets were preprocessed as part of a previous study and reused in the present work; for preprocessing procedures, see Ref.^75^. The final datasets consisted of perturbed genes, referenced against the GRCh38 genome, presented as log_2_ fold-change expression matrices between perturbed and non-perturbed experiments. To the best of our knowledge, this is the only dataset including expression of perturbed RBPs in liver cancer where matched eCLIP-seq studies are available.

### Binding sites detection with eCLIP-seq and RAP-seq

To analyze and validate the RBP regulome, we utilized positive control data for enhanced crosslinking and immunoprecipitation sequencing (eCLIP-seq)^76^ from ENCODE and RNA affinity purification followed by sequencing (RAP-seq) from the in-house repository^28,56^. Both methods enable transcriptome-wide profiling of RBP binding sites, albeit with procedural differences. The main difference between these two techniques is their experimental protocol^56^ since eCLIP-seq is performed *in cellulo* while RAP-seq is conducted in vitro. The number of profiled RBPs is much larger for eCLIP-seq (n = 103) than for RAP-seq (n = 9) experiments, considering RBP-RNA interactions significant for peaks with a *p*-value (*P*) < 0.05. In total 106 sets of RBP-RNA binding sites from the combined eCLIP-seq and RAP-seq datasets of HepG2 were used as positive controls for validation. These datasets were selected based on the availability of experimentally derived RBP–RNA binding data in the same cellular context as the inferred GRNs. We utilized eCLIP-seq data from ENCODE and added in-house RAP-seq data generated with HepG2 cells, as these were directly available and compatible with the shRNA-seq data used for GRN inference. Together, these datasets provide robust experimental support for the predicted RBP–RNA interactions included in this study.

### ENCODE-based synthetic data and benchmarking

To generate ENCODE-like synthetic datasets supplied with gold standard GRNs, we utilized GeneSPIDER^26^ aiming at inference and benchmarking with controlled GRN and data properties (publicly available at bitbucket.org/sonnhammergrni/genespider). In the simulation, we assumed the same data properties as the real ENCODE shRNA RNA-seq, i.e. data size of 232 RBPs and two replicates. In addition, based on Fig. S1B, GRN was generated with the average outgoing node degree of 3 (excluding the self-loops). To find the synthetic data set that is the most similar to ENCODE, we iteratively simulated gene expression data with various signal-to-noise ratios (SNRs) and GRNs. In the first step, we started by drawing $SNR\_L$ between 0.0001 and 0.1 and calculated the Pearson correlation coefficient (Matlab function *corrcoef*) among replicate pairs. The SNR_L is defined as follows:

1 $$SNR\_L =\frac{{\Sigma}_{min}(X)}{\sqrt{{\chi }^{-2}(\alpha , N\times M){\sigma }^{2}}}$$

where ${\Sigma}_{min}(X)$ is the smallest singular value of the noise-free gene expression matrix $X$, ${\chi }^{-2}(\alpha , N\times M)$ is the inverse ${\chi }^{2}$ distribution at level $\alpha$ with $N\times M$ degrees of freedom (number of genes $\times$ number of experiments), and ${\sigma }^{2}$ is the variance of the additive noise matrix. The synthetic gene expression was generated as steady state data ($Y$) following the linear mapping: $Y=-{A}^{-1}P + E$, where $A$ is an adjacency matrix of a GRN, with negative real part for eigenvalues, $P$ is the matrix of the perturbation design, and $E$ is the matrix of Gaussian noise defined at the $SNR\_L$ level. To mimic the ENCODE data $P$ is a diagonal matrix where -1 indicates the knockdown of a given gene and 0 indicates unperturbed genes. Next, we compared average correlation ${\mu}_{\rho \_E}$ of simulated data to the average correlation ${\mu}_{\rho \_S}$ of the real ENCODE data as ${\mu}_{\rho \_diff}=\left|{\mu}_{\rho \_E}-{\mu}_{\rho \_S}\right|$. The procedure was executed 1000 times for 20 randomly generated scale-free GRNs. The corresponding GRN and data with the lowest ${\mu}_{\rho \_diff}$ were kept as the closest to the real data. Specifically, we selected synthetic data for HepG2 and K562 with ${\mu}_{\rho \_diff}=$ 1.62e−05 and ${\mu}_{\rho \_diff}=$ 1.73e−04, respectively. This corresponded to $SNR\_L$=0.0055 for HepG2 and $SNR\_L$=0.0029 for K562. The synthetic ENCODE-like datasets generated in this manner, along with their corresponding gold standard GRNs, were used for benchmarking.

The ENCODE-like data was used to perform benchmarking on the consensus GRN approach and tune the methodology towards the real ENCODE data. To assess the performance of the consensus-based approach and investigate how many true positive links are kept, we used a positive predictive value ($PPV$) measure:

2 $$PPV = \frac{TP}{TP+FP}$$

where TP is the number of true positive links and FP is the number of false positive links. We also used more global performance metrics such as the F1 score, which is the harmonic mean of precision and recall:

3 $$F1 score = \frac{2\times TP}{2\times TP+FP+FN}$$

where FN is the number of false negative links.

### Inference of consensus GRNs

To infer a consensus GRN, we employed the following 10 methods: normalized least squares (LSCON)^77^, ridge regression with least square cutoff (ridgeco), total least squares with cut-off (tlsco), lasso, logistic regression with lasso (loglasso), classification with support vector machine (svmc), z-score (Zscore), decision trees (CART), regression neural networks (neunetreg) and linear gaussian processes (gaplin) included in the GeneSPIDER framework^26^. These methods have been efficiently used in previous studies and analyses^27,78,79^. Recently introduced methods in the framework, including decision trees and neural network regression, have also been successfully applied for the GRN inference^71,80,81^. Notably, benchmarking revealed that two methods were redundant, so they were excluded. Namely, least squares, due to its tendency to overestimate the GRN, and the elastic net, as it performed slightly worse than lasso while obtaining almost identical GRN.

To construct consensus GRNs, we used the WOC approach, in which each potential link was assigned a weight reflecting the number of inference methods that predicted it, divided by the total number of methods. For example, a weight of 0.5 indicates that five out of ten methods inferred the link. Links consistently detected across multiple inference methods were retained, whereas conflicting predictions (i.e., interactions supported inconsistently across methods) received lower consensus scores due to reduced agreement among methods in the consensus framework. Consequently, these interactions were conditionally excluded during the network construction if their consensus support did not meet the selected threshold. Here, we define a network including links supported by at least *n* methods as an *n* + consensus GRN, with the 3 + GRN serving as our primary focus. This threshold was guided by benchmarking on synthetic HepG2-like data, where the 3 + GRN achieved the highest F1-score while maintaining high precision (Fig. S2H-I). The HepG2 3 + GRN derived from bulk data includes 226 RBPs and 648 interactions, with an average node degree of three, consistent with the gold-standard GRNs (Fig. S1B and S4G). Higher consensus thresholds produced sparser networks with fewer potential FPs (Fig. S2 and S3), whereas a 2 + GRN was used selectively to validate targets within highly connected RBP hubs (Fig. 1B–C), based on benchmarking that showed improved precision even with links inferred by only two methods (Fig. S2D–E). Overall, to optimize GRN pruning, we adopted two complementary strategies: emphasizing high-confidence interactions in sparse GRNs (5 +), while also validating denser GRNs (2 + or 3 +) to capture more potential true positives.

The sign of the links, indicating inhibition or activation, in the consensus GRN was established by using the WOC approach as well. The sign was established based only on inference methods capable of sign estimation, i.e. all methods excluding CART and neunetreg. We complemented the consensus of sign-inference methods by incorporating Spearman correlation coefficients ($\rho$), computed for each interaction using averaged ENCODE expression data. Importantly, for indecisive cases, e.g. when two methods inferred negative sign and two others positive sign, the sign was taken only based on the $\rho$.

### Validation of consensus GRN

To validate the 3 + consensus GRN, we used several resources and external data sets to gather information about positive controls for liver cancer (Table 2). These included: (1) gene expression from 110 healthy liver tissue samples from Genotype-Tissue Expression (GTEx) project, (2) gene expression from 421 samples from Liver Hepatocellular Carcinoma (LIHC) samples from The Cancer Genome Atlas (TCGA), and (3) clinical data with survival statistics of LIHC from TCGA. For survival data, we utilized a log-rank test and calculated the *P* value for each RBP that was included in the GRN. To combine the pairs of *P* values obtained for each gene within an interaction, we used harmonic mean *P* value. We support our selection of GTEx and TCGA cohorts due to their status as the most comprehensive and well-established cancer gene expression datasets. To perform a fair comparison, unified UCSC Xena cohorts with transcripts per million (TPM) gene expression values were used for the former mentioned three datasets^82^. GTEx and TCGA cohorts were employed to calculate Pearson’s correlation coefficient of LIHC and healthy liver tissue, together with the corresponding *P* value. The values given in the validation tables represent the difference in the coefficients between LIHC and healthy liver tissue. Additionally, (4) from EuRBPDB, we incorporated literature mentions of RBPs related to cancers and differential expression of RBPs detected in case–control studies of cancer into a “cancer literature popularity score”^83^. (5) we also used ENCODE-derived eCLIP-seq data for HepG2 and K562 cells, representing a landscape of RBPs-RNA interactions, annotated with GENCODE v45^84^ using the *queryGff* R function with default settings from the RCAS package (v1.22.0). These eCLIP-seq data were merged with significant peaks (FDR < 0.05) from the RAP-seq dataset (22). (6) An in-house RBP list and an estimated number of literature mentions of genes being RBPs^85^ were also included. Furthermore, (7) we included a functional association network for human from FunCoup (v5), aimed at detecting direct and indirect functional associations based on regulatory mechanisms or pathways, constructed using 10 evidence types, including genomics, proteomics, and transcriptomics data^86^. We provide reasoning for choosing FunCoup, as it is a global and comprehensive functional association network that captures multi-omics data and has demonstrated superior performance compared to databases like STRING and HumanNet^86,87^. Here, discrete association scores absent in FunCoup were predicted using decision trees, with a cross-validation accuracy of 85%, applied to tabular data including all the other validation features (points 1–6) used as a training set. Decision trees were chosen for their efficiency in learning from tabular data, fast performance, handling of missing data, and the learning process is interpretable^88^. Training and testing were performed using the rpart package (v4.1.19).

**Table 2:** An overview of the validation data sets. RBPs coverage is given by intersecting the RBP list included in the ENCODE shRNA-seq data and given resources. Links and RBPs coverage is given by intersecting links or RBPs from the 3 + consensus GRN. For RBPs coverage of 1% or smaller, names of RBPs were given instead of the percentage value.

Resource	Data type	Samples	RBPs coverage	Links coverage
GTEx	Bulk RNA-seq	Patients: healthy liver	100%	100%
TCGA	Bulk RNA-seq	Patients: LIHC	100%	100%
ENCODE	eCLIP-seq	Cell line: HepG2	35%	63%
in-house	RAP-seq	Cell line: HepG2	4%	8%
GRAND	Bulk RNA-seq	Cell lines (LIHC): JHH6, SNU398, SNU886, SKHEP1, HLF, SNU449, SNU475, LI7, HUH1, JHH4, SNU387, HUH7, SNU182, SNU423, SNU761, JHH2, JHH1, HEP3B217, HUH6, SNU878, JHH5, HEPG2, JHH7, PLCPRF5	*CEBPZ*, *LIN28B*, *YBX3*	4%
		Patients: TCGA (LIHC)	*CEBPZ*	2%
GRNdb	Single-cell RNA-seq	Cells: Liver cancer (GSE98638)	CEBPZ, NELFE, BCLAF1, GTF2F1	2%
		Cells: Healthy liver (GSE98638, GSE134355)	*CEBPZ*, *NELFE*, *BCLAF1*	1%
FunCoup	Multiomics	Healthy tissue	82%	58%

To obtain a comprehensive table including all categorical features, non-categorical variables were clustered using parameterized finite Gaussian mixture models (GMM) (Mclust function with default settings, from the mclust v6.1 R package) into three groups. We motivate the choice of three groups, by evaluating various levels based on the performance of decision trees and for the sake of interpretability. In the case of having multiple zero values in a variable, GMM was applied to non-zero values to obtain two groups, and a zero value was taken as the third group. This approach was applied for variables including co-expression difference, literature-related features, and signal values from eCLIP-seq and RAP-seq. Other features, such as survival significance group, differential expression significance group, consensus frequency, and presence in the K562 GRN and RBD group were treated as categorical variables. Next, all features were scaled between 0 and 1 using min–max normalization to standardize scores across them. To estimate the total score, we calculated the average of the scaled feature scores for each interaction, thereby assigning equal weight to all features, and subsequently sorted the table in descending order.

To obtain a set of interactions significantly related to liver cancer-related terms, an enrichment analysis was performed on sets of RBSs by incrementally selecting the top interactions in the top-down direction from the *P* value sorted list. This enrichment analysis was performed with the human disease-associated gene sets from DisGeNET using a hypergeometric test with the *enrichDGN* function (default settings) from the DOSE package (v3.22.1)^89^. We selected terms and their *P* values where “liver” or “hepato” phrases, in the context of cancer, were present. We then adjusted *P* values for FDR within each run and provided an averaged FDR value calculated as the harmonic mean of all terms (R package harmonicmeanp v3.0.1). The optimal number of links was selected based on the enrichment with the lowest average FDR estimated as the global maximum of -log_10_(*P*) based on Fig. S5. This allowed us to establish a set of interactions with high validation scores and related to liver cancer.

### Validation of TFs with external GRNs

In a separate part of our analysis, we analyzed a set of TFs included as a part of our data. We incorporated several bulk and single-cell RNA-seq-based GRNs from GRAND and GRNdb^90,91^ From GRAND, we collected liver cancer-related GRNs corresponding to 24 different cell lines and one set of TCGA liver-cancer patients, estimated with LIONESS and OTTER, respectively^92^. From GRNdb, we used TF GRNs of human liver-related cancers derived from single-cell data^91^. In GRNdb, GRNs were inferred using GENIE3^80^. The GRNs from GRAND and GRNdb were intersected with our 3 + consensus GRN.

### Module detection and enrichment analysis

To detect modules on GRNs, we applied the igraph (1.4.1) function *cluster_infomap* that minimizes the expected description length of a random walker trajectory^93^ as benchmarks showed their good performance^94^. Initially, we identified clusters in GRN to filter out disconnected nodes. Subsequently, modules containing five or more genes were retained for further analysis. Then, we reapplied the detection of modules on the filtered GRN using the same approach. This allowed us to get much cleaner GRN and larger modules for the enrichment analysis. Next, we employed the clusterProfiler (4.4.4) package and its *enricher* function to perform a hypergeometric test and assess the significance of the overlap between modules and the MSigDB gene sets^95,96^. Specifically, we selected curated (C2) and hallmark (H) gene sets, using an in-house RBP list as a background^85^. Afterward, we collected *P* values for each module and processed the results by (1) removing terms enriched with only one gene, (2) performing module-oriented FDR adjustment, and (3) keeping only terms with significant FDR values (FDR < 0.05).

### Drug repurposing on the selected targets

To identify potential drug candidates for liver cancer treatment, we performed in silico drug repurposing through three approaches. First, we submitted the entire signed 5 + consensus GRN to the CLUEreg tool^90^. Second, we provided target sets for master regulators to CLUEreg. We selected all targets of IGF2BP1 based on eCLIP-seq and RAP-seq, further limited to include the top 20% of differentially expressed genes (DEGs) based on Student’s t-test using TCGA and GTEx liver-related cohorts. Additionally, based on Fig. 1B, we investigated all targets of AQR and U2AF2 based on 2 + consensus GRN, similarly limited to the top 20% of DEGs. As CLUEreg requires lists of down- and up-regulated targets as low- and high-targeted genes respectively, we utilized the aforementioned information about DEGs. The drugs were selected based on their high cosine similarity and low tau from the top 100 estimated by CLUEreg.

The third approach involved intersecting lists of selected RBPs with the Comparative Toxicogenomics Database (CTD)^97^. Specifically, we used CTD to identify *MYC*-related targets identified through module enrichment analysis (Table 1) and a list of the most noteworthy interactions (Table S5) with their co-expression changes (Fig. S12). The output of CTD was then intersected with DrugBank to compile a list of drugs^98^. Finally, we retained drugs that interacted with 50% or more of the RBPs in the specified set.

## Supplementary Information

Below is the link to the electronic supplementary material.

Supplementary Material 1

Supplementary Material 2

Supplementary Material 3

Supplementary Material 4

## Data Availability

Simulated ENCODE-like datasets are publicly available via Zenodo at https://zenodo.org/records/12165429. HepG2 and K562 shRNA-seq and eCLIP-seq datasets used in this study are publicly available through the ENCODE project repository (https://www.encodeproject.org/). RAP-seq datasets are publicly available through ArrayExpress under accession number E-MTAB-10834.

## Code availability

GRN inference was performed using the GeneSPIDER MATLAB package, which is publicly available at https://bitbucket.org/sonnhammergrni/genespider/. All downstream analyses presented in this study were performed in R, and the corresponding scripts have been made publicly available for reproducibility through GitHub and archived in Zenodo at https://zenodo.org/records/20316430.
