# Supplementary material for: Comprehensive analysis of the RBP regulome reveals functional modules and drug candidates in liver cancer
Source: Sci Rep. 2026 Jun 26;16:19626. doi: 10.1038/s41598-026-58864-6 (PMC13309532; doi:10.1038/s41598-026-58864-6)
Supplement: Supplementary file 4 — Supplementary Material 4 [file 41598_2026_58864_MOESM4_ESM.pdf]

Supplementary material

Comprehensive analysis of the RBP regulome reveals functional modules and drug candidates in liver cancer

Mateusz Garbulowski[1,2,*], Riccardo Mosca[3], Carlos J. Gallardo-Dodd[3], Claudia Kutter[3], Erik L. L. Sonnhammer[1,*]

[1]Department of Biochemistry and Biophysics, Stockholm University, Science for Life Laboratory, Solna, Sweden

[2]Department of Immunology, Genetics and Pathology, Uppsala University, Uppsala, Sweden

[3]Department of Microbiology, Tumor, and Cell Biology, Karolinska Institute, Science for Life Laboratory, Solna, Sweden

Supplementary methods 2
    Highly precise GRN of RBPs in liver cancer 2
    Packages used for the analysis 2
Supplementary figures 3
Supplementary tables 17
Supplementary references 21

Supplementary methods

Highly precise GRN of RBPs in liver cancer

To infer a reliable GRN of RBPs for liver cancer cells, we applied and benchmarked several GRN inference methods to develop a consensus approach (**Figure S1, S2, and S3**). To evaluate the consensus GRN, we applied the approach to ENCODE-like synthetic data including gold standard GRNs. First, we investigated the performance of each method alone, which in general was poor (**Figure S2 and S3 A-B**) as the ENCODE-like data was simulated with low SNR. Next, we investigated distance-based similarity across inferred GRNs (**Figure S2C, S3C**). The results of clustering show diversity in terms of GRN topology across all methods and also similarity for certain subgroups of methods. For example, decision trees and neural networks are clustered together as methods that infer the most similar GRNs. However, none of the methods inferred a highly similar GRN to the gold standard (**Figure S2C, S3C**). For real ENCODE data sets, we can see a similar behavior in terms of distances between methods and their contributions to consensus links (**Figure S4**). Moreover, we performed a pairwise comparison of the inference methods (F**igure S2, S3 D-F**). We calculated the $\log_2$ fold-change ($\log_2$FC) as a ratio of PPV from a pairwise consensus over PPV from single GRNs. We can observe that the majority of pairwise intersections result in improvement, i.e. $\text{Log}_2\text{FC} > 1$ (**Figure S2D, S3D**). We also collected all the PPVs from pairwise comparisons and compared them with PPVs from single GRNs (**Figure S2, S3E**). The precision between consensus and single GRNs is significantly (Student's t-test $P < 0.05$) higher for the pairwise consensus approach. Next, we investigated the density of TPs and FPs lost due to pairwise consensus comparison (**Figure S2, S3 F**). The results show that more FP links are removed than TP links when using a consensus approach. To investigate which and how many inference methods participated in discovering links for all consensus thresholds, we inspected their amount for each method (**Figure S2G, S3G**). In the majority of cases, it showed a balanced proportion of inferred links. Similar trends are visible for the real ENCODE data sets (**Figure S4**). To investigate the overall correctness of all consensus thresholds, we measured the PPVs and F1 scores by pruning the links for every possible minimum number of consensus links (**Figure S2, S3 H-I**). Lastly, we evaluated all possible consensus GRNs in terms of their degree and F1 score (**Figure S2H, S3H**). We observed that the most accurate GRN in terms of F1 score was created for 3+ consensus links. However, any GRN with more than three consensus links has a higher PPV with the cost of having fewer links (**Figure S2I, S3I**).

Packages used for the analysis

Several R libraries were used for the analysis and visualization. Harmonic mean of *P* values was calculated using the harmonicmeanp (v3.0.1) library with a function *p.hmp*. For processing and visualization of RAPseq and eCLIP-seq, we applied rtracklayer (v1.56.1), IRanges (v2.30.1) and GenomicRanges (v1.48.0) packages. Survival analysis was performed with the function *survfit* from the survival (v3.4-0) R package. For binning continuous validation features, mclust (v6.1) R package for Gaussian Mixtures modeling was utilized. The R.matlab package (v3.7.0) allowed to handle GeneSPIDER output in R. Finally, viuzaliation of the results was performed with various packages: pheatmap (v1.0.12), ggplot2 (v3.5.0), ggraph (v2.1.0), ggnet (v0.1.0), ggpubr (0.6.0) and ggrepel (v0.9.4).

Supplementary figures

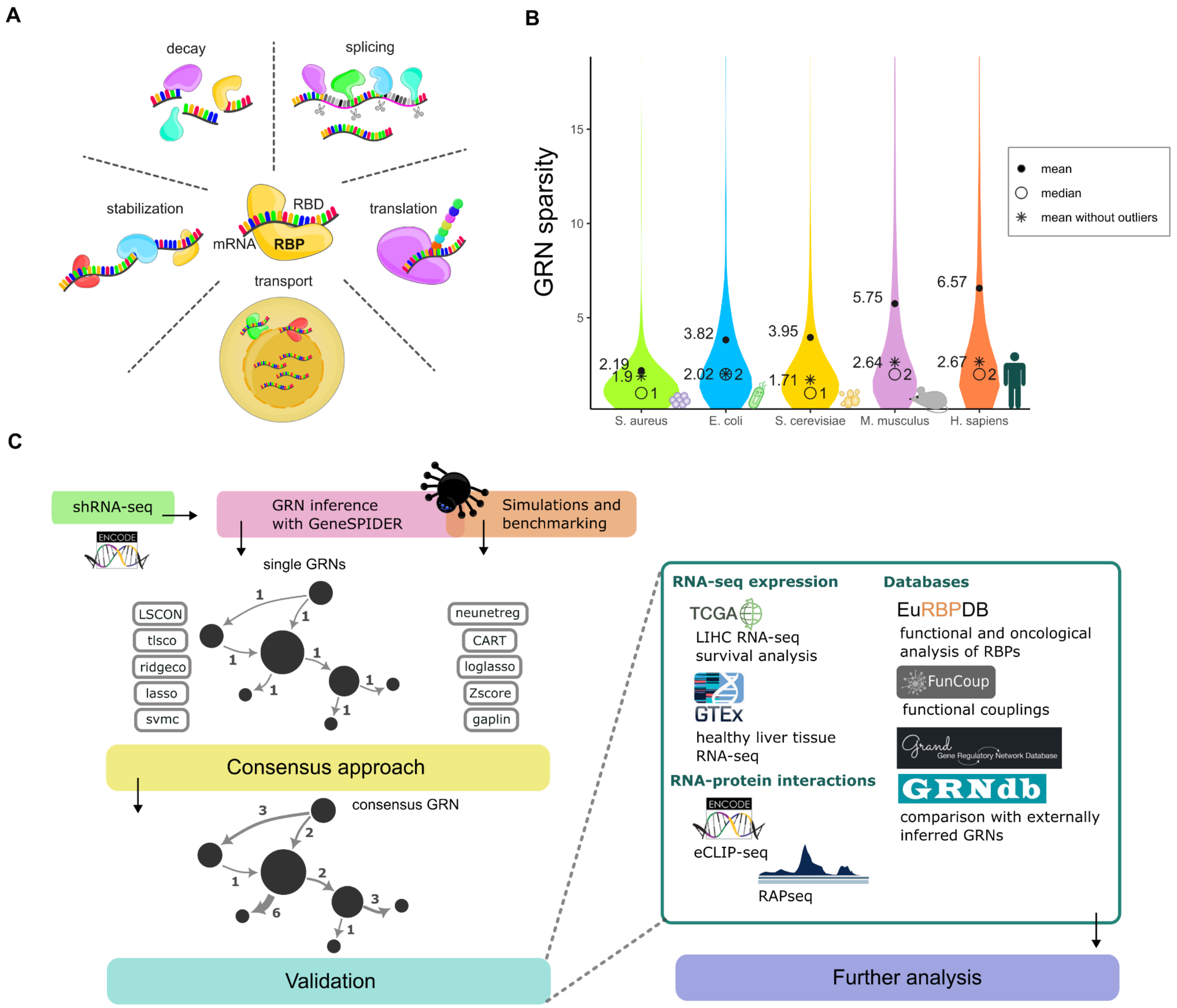

Figure S1. Overview of the GRN study on RBPs **A.** RBP functions in cells presenting their important role in posttranscriptional processes **B.** Violin plot shows GRN degree (sparsity) distributions estimated based on gold standard biological networks from DREAM5 challenge and TRRUST in five species (color-coded). Outliers were removed. **C.** General pipeline of consensus approach and validation chosen in this study. GRN inference methods used for the consensus approach and all external resources used for the validation are listed. Further analysis includes functional modules analysis and drug repurposing.

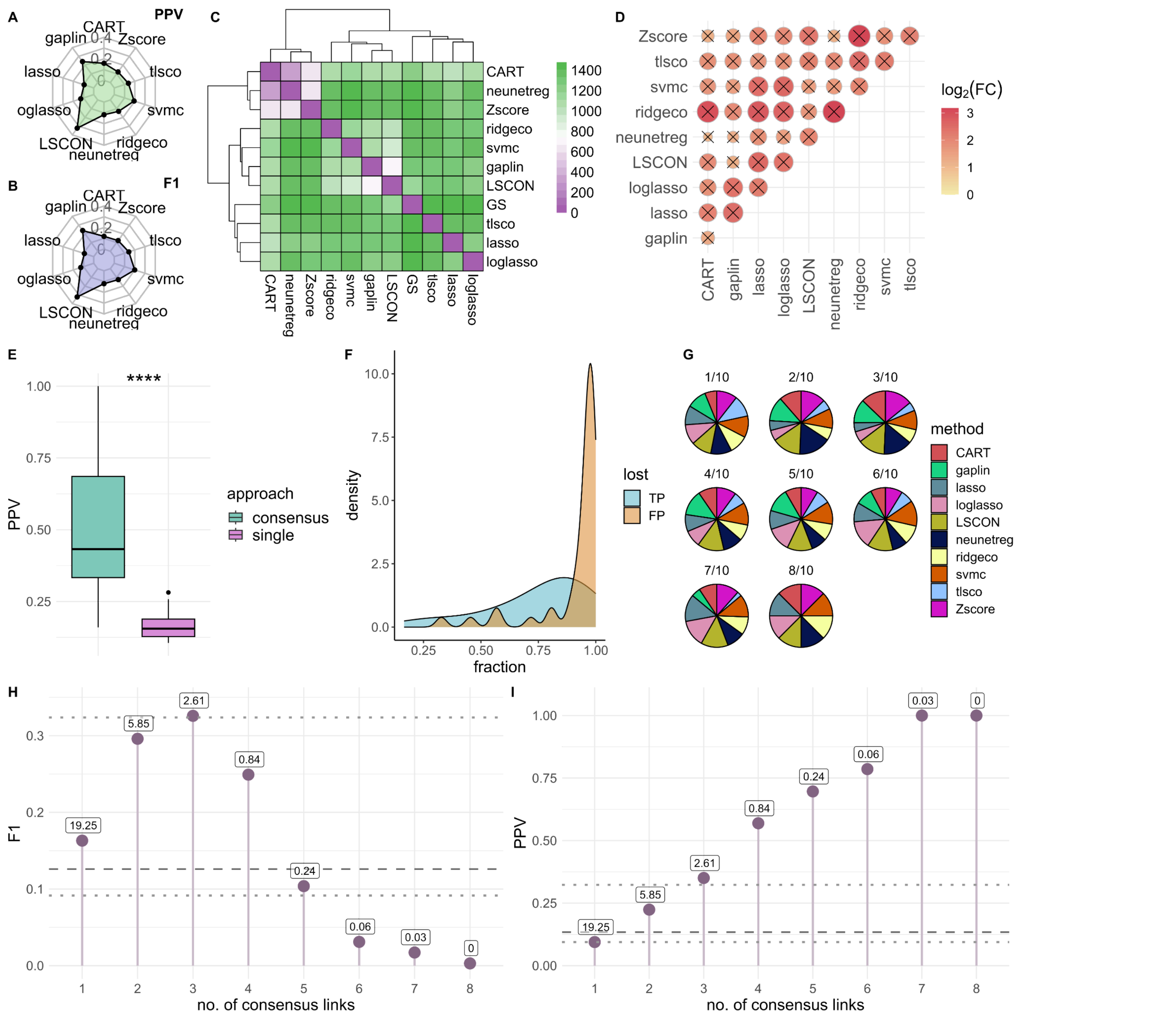

Figure S2. Summary of benchmarking synthetic HepG2-like data generated with SNR_L fitted to real HepG2 data. Diagonal (selfloops) was excluded from calculating benchmarking metrics in all subplots. **A-B.** Radial plots show (**A**) positive predictive values (PPV) and (**B**) F1-score (F1) values of each inference method used in this study. **C.** Heatmap displays Hamming distances between inferred GRNs (low: purple, high: green). **D.** Circle plot indicates pairwise consensus approach precision fold-change (FC_{PPV}), i.e. $\frac{PPV_{consensus}}{PPV_{single}}$, of obtained networks. The diameter indicates the $\mathrm{Log_2FC}$ value. Circle color displays fold-changes (low: yellow, high: red). Cross denotes whether a $log_2\left(FC_{PPV}\right)$ is greater than 1. **E.** Boxes indicate PPV values for each method employed based on the consensus (green) and single approach (purple). Boxes delineate the interquartile range (IQR) and the median (horizontal line), and whiskers indicate upper or lower quartile plus or minus 1.5 × IQR, respectively. Stars (****) indicate $p \leq 0.0001$ from Student's *t*-test. **F.** Density plot displays a fraction of lost true positive (TP) versus false positive (FP) links in pairwise consensus approach **G.** Piecharts show proportional contributions of true positive links by all methods for a given consensus threshold **H-I.** Line plots show the effect of consensus thresholds on PPV and F1-score. In the label above each point, the overall network degree is given. Lines denote min (dotted), max (dotted), and median (dashed) obtained across single networks.

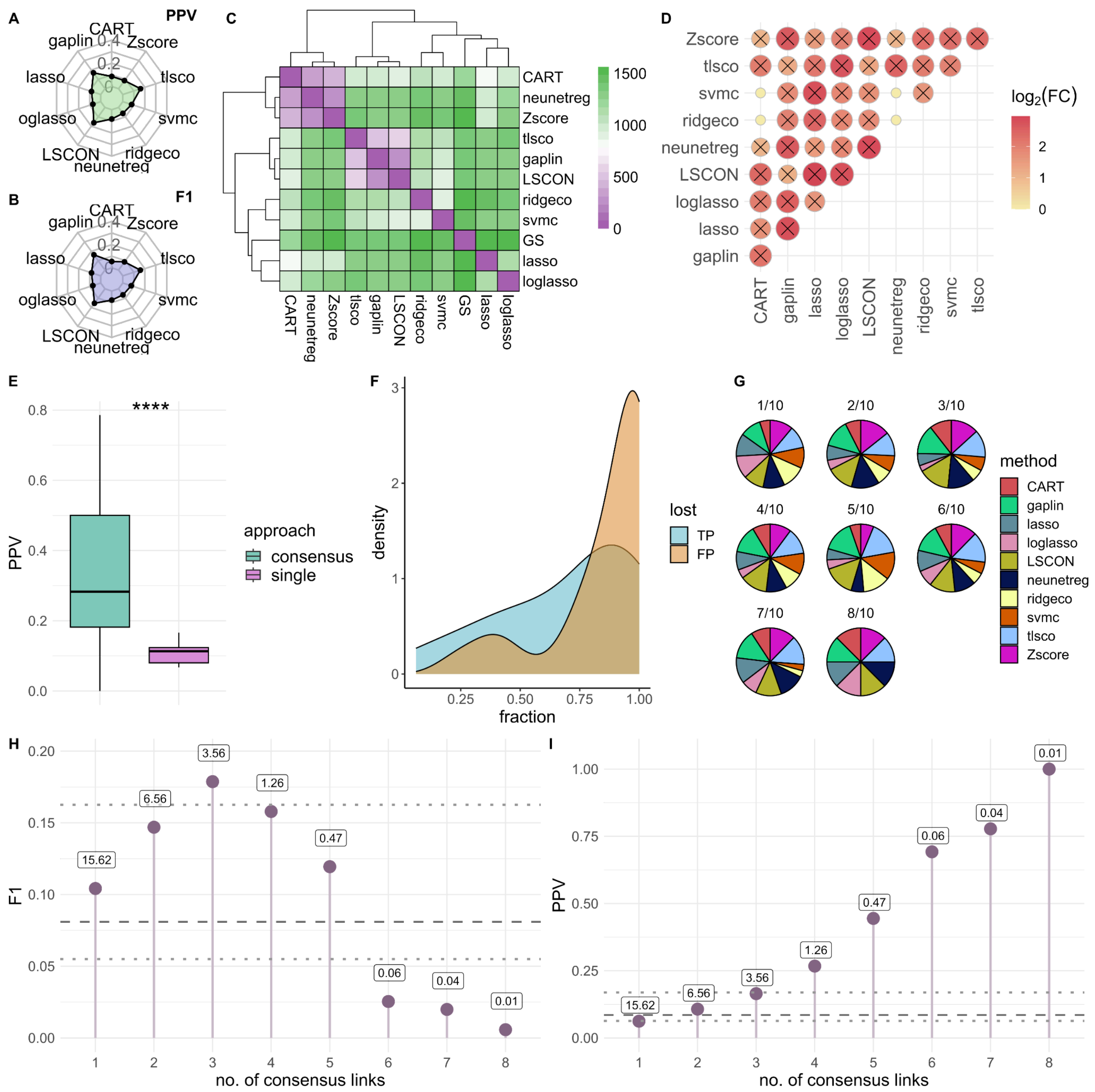

Figure S3. Summary of benchmarking synthetic K562-like data generated with SNR_L fitted to real K562 data. Diagonal (selfloops) was excluded from calculating benchmarking metrics in all subplots. **A-B.** Radial plots show (**A**) positive predictive values (PPV) and (**B**) F1-score (F1) values of each inference method used in this study. **C.** Heatmap displays Hamming distances between inferred GRNs (low: purple, high: green). **D.** Circle plot indicates pairwise consensus approach precision fold-change (FC_{PPV}), i.e. $\frac{PPV_{consensus}}{PPV_{single}}$, of obtained networks. The diameter indicates the Log$_2$FC value. Circle color displays fold-changes (low: yellow, high: red). Cross denotes whether a $log_2\left(FC_{PPV}\right)$ is greater than 1. **E.** Boxes indicate PPV values for each method employed based on the consensus (green) and single approach (purple). Boxes delineate the interquartile range (IQR) and the median (horizontal line), and whiskers indicate upper or lower quartile plus or minus 1.5 × IQR, respectively. Stars (****) indicate $p \leq 0.0001$ from Student's *t*-test. **F.** Density plot displays a fraction of lost true positive (TP) versus false positive (FP) links in pairwise consensus approach **G.** Piecharts show proportional contributions of true positive links by all methods for a given consensus threshold **H-I.** Line plots show the effect of consensus thresholds on PPV and F1-score. In the label above each point, the overall network degree is given. Lines denote min (dotted), max (dotted), and median (dashed) obtained across single networks.

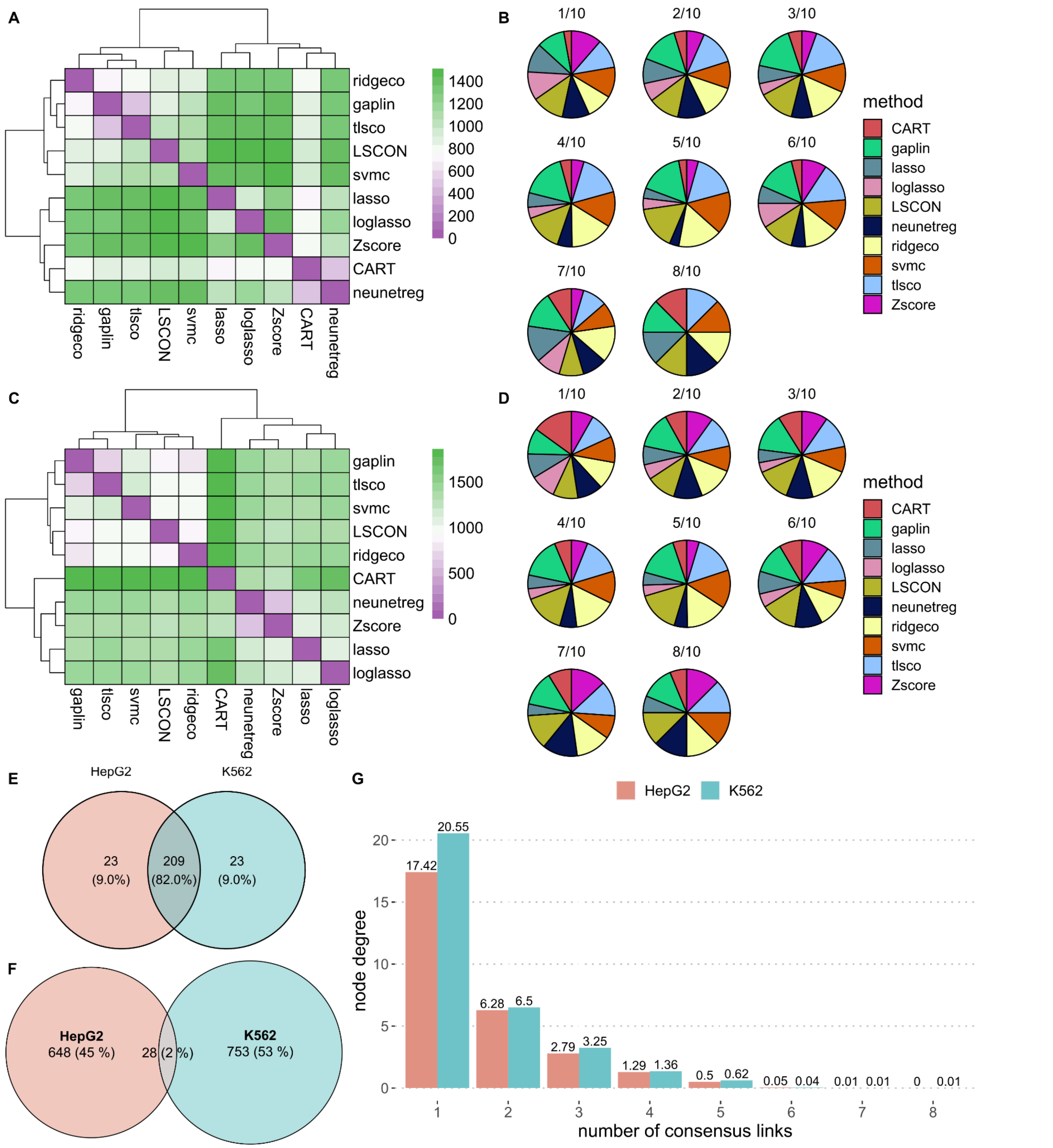

Figure S4. Summary statistics of the ENCODE data and inferred networks. **A.** Hamming distances between inferred GRNs for HepG2-like data **B.** Contribution of true positive links by all methods for a given consensus threshold for HepG2-like data **C.** Hamming distances between inferred GRNs for K562-like data **D.** Contribution of true positive links by all methods for a given consensus threshold for K562-like data **E.** Total number of RBPs in both cell lines and their common part **F.** Common links detected between both cell lines for at least 3 consensus links (3+). **G.** Average node degree of consensus networks for a given number of consensus links used as a threshold for pruning GRN.

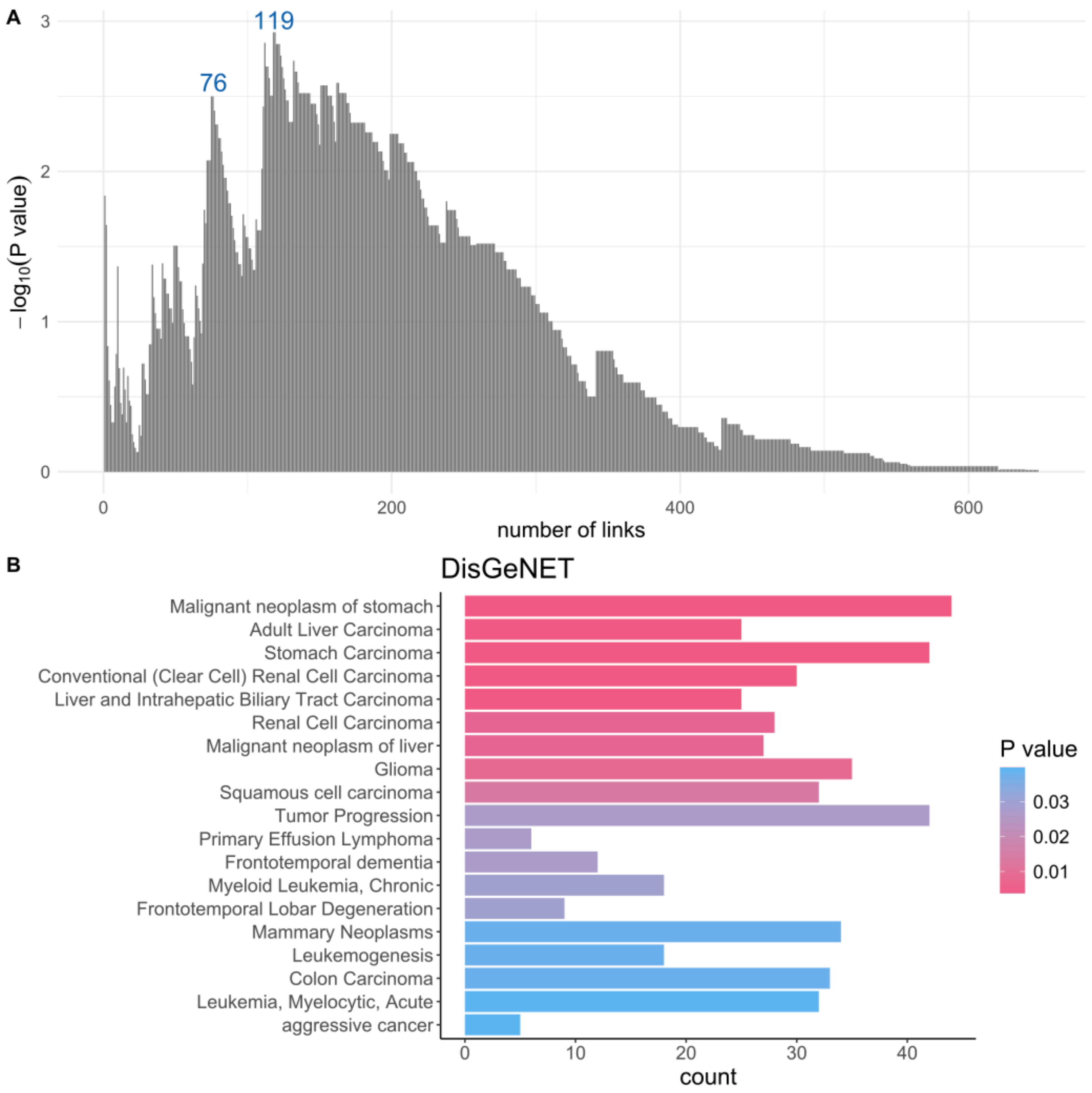

Figure S5. Estimating the threshold for interactions significantly related to liver cancer-related terms. **A.** Incremental threshold moving top-down through a list of interactions decreasingly sorted by validation score. Local maxima were estimated with a span of 30 and marked above each peak. **B.** DisGeNET enrichment for genes selected from interactions where enrichment *P* value was the lowest (topmost 119). *P* values were adjusted for False Discovery Rate (FDR). DisGeNET terms are shown for FDR ≤ 0.05.

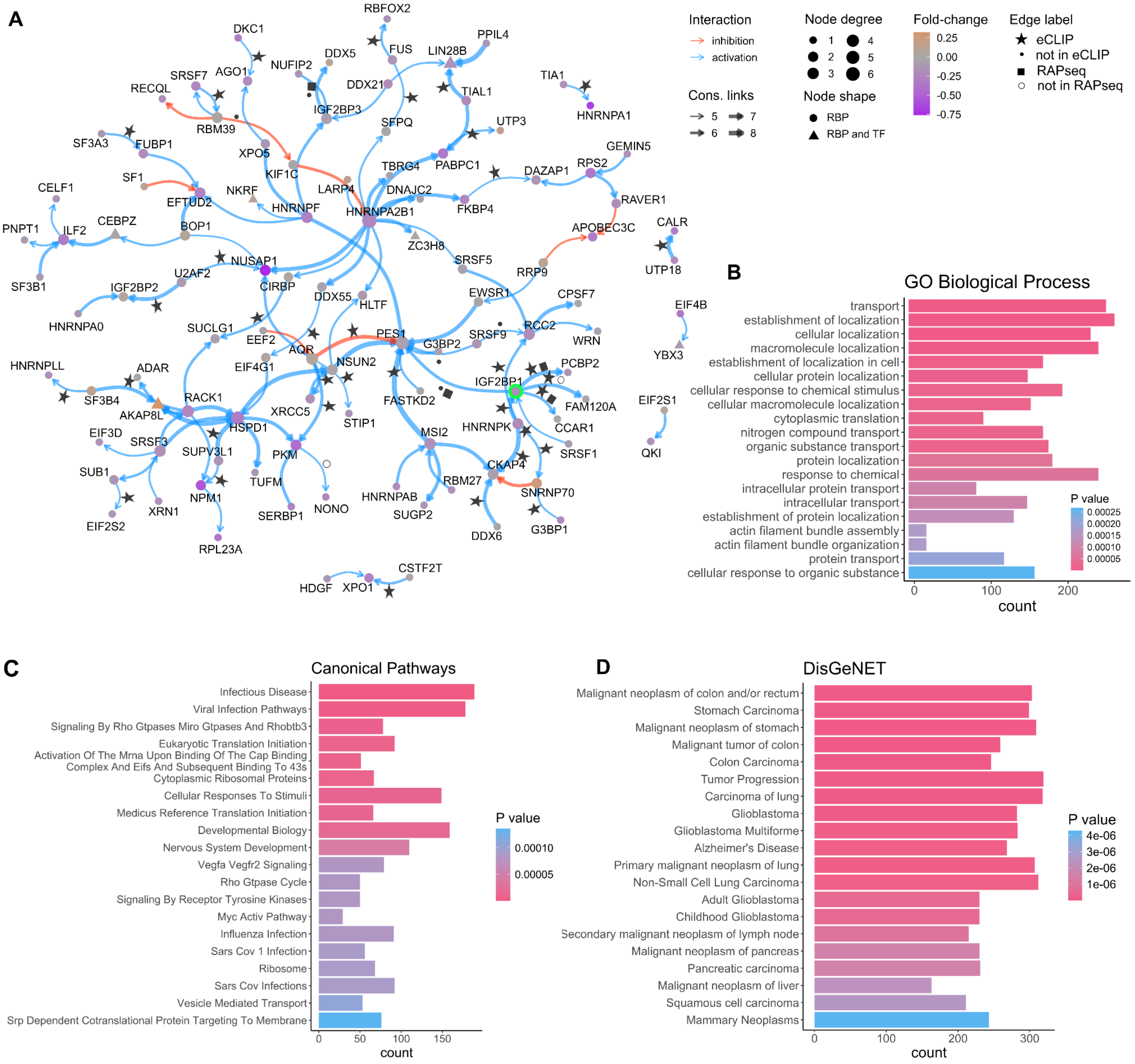

Figure S6. A. GRN of topmost 119 validated interactions that correspond to enrichment towards liver cancer. *IGF2BP1* is highlighted (node with green border) as an RBP regulator corroborated by eCLIP-seq and RAPseq data. **B-D.** Enrichment analysis of *IGF2BP1* targets taken together from eCLIP-seq and RAPseq. The cutoff for *P* value was set to 0.01 and FDR adjustment was applied. The plots show the top 20 terms.

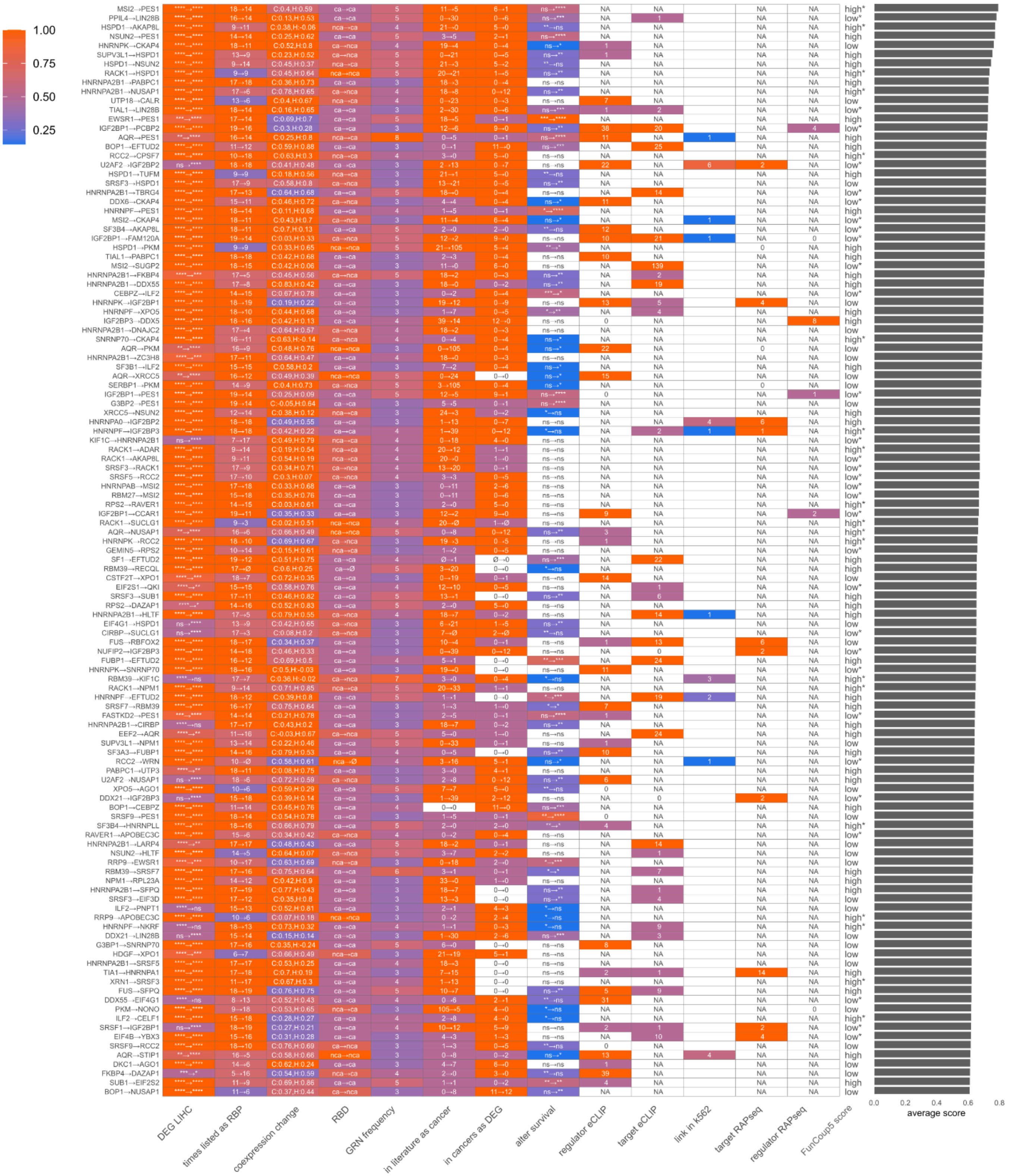

Figure S7. Summary of the consensus GRN validation for the top 119 links, selected as a global maximum of enriching liver cancer-related terms from DisGeNET (**Figure S5**). In addition, stars indicate significance levels such as $p \leq 0.001$ (****), $p \leq 0.01$ (***), $p \leq 0.05$ (**), $p \leq 0.1$ (*) and $p > 0.1$ (ns). This table is a subtable of **Supplementary Table S3**. Co-expression change is a difference between Pearson's correlation coefficients in LIHC and GTEx. Scores are scaled between 0 and 1. Some abbreviations were marked on the plot: cancer (C), healthy (H), canonical (ca), non-canonical (nca), binding domain (RBD) and differentially expressed genes (DEG).

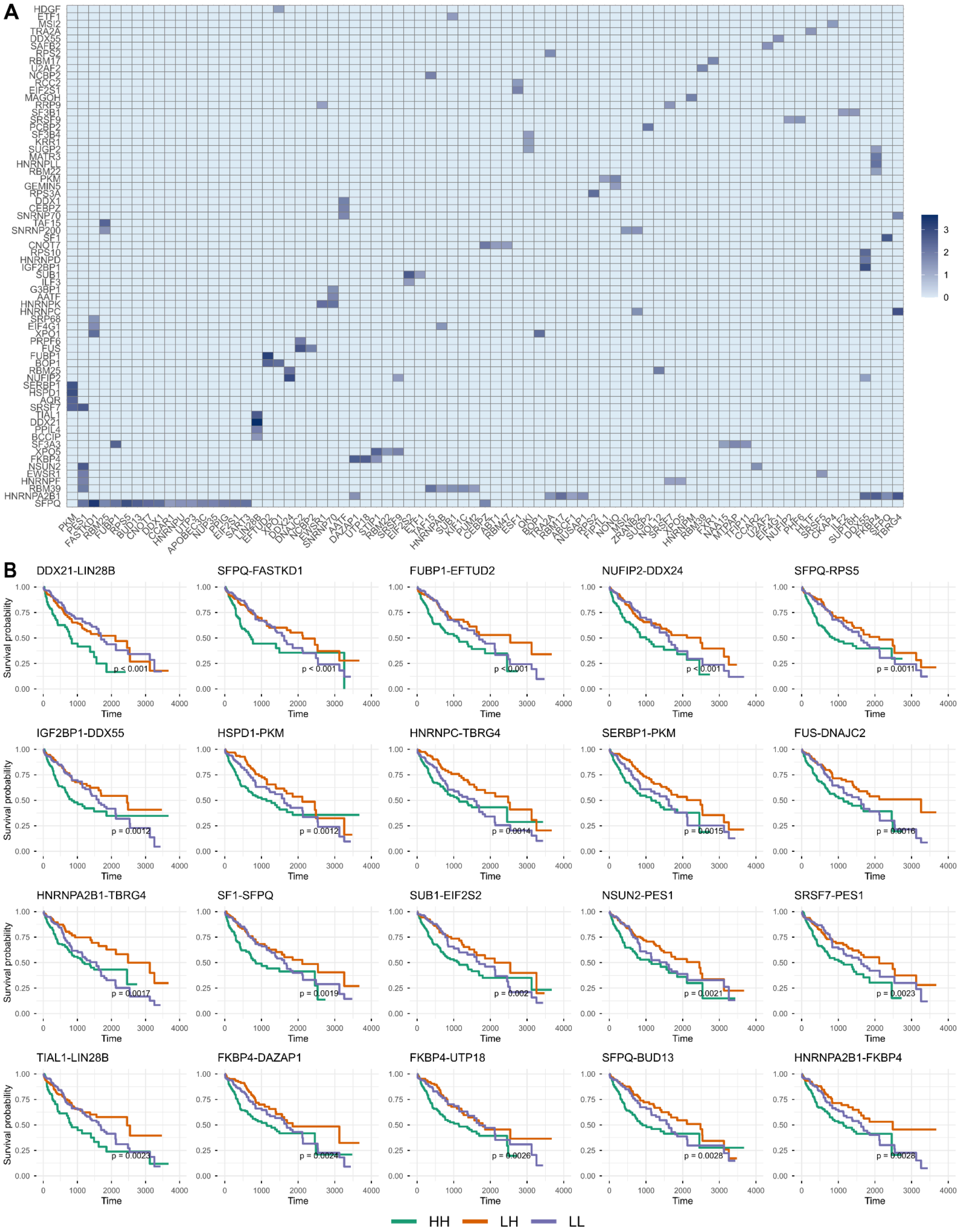

Figure S8. Survival analysis of full 3+ GRN (648 interactions) where **A.** only significant (P < 0.05) influence on survival is shown and **B.** top 20 interactions affecting survival with lowest P.

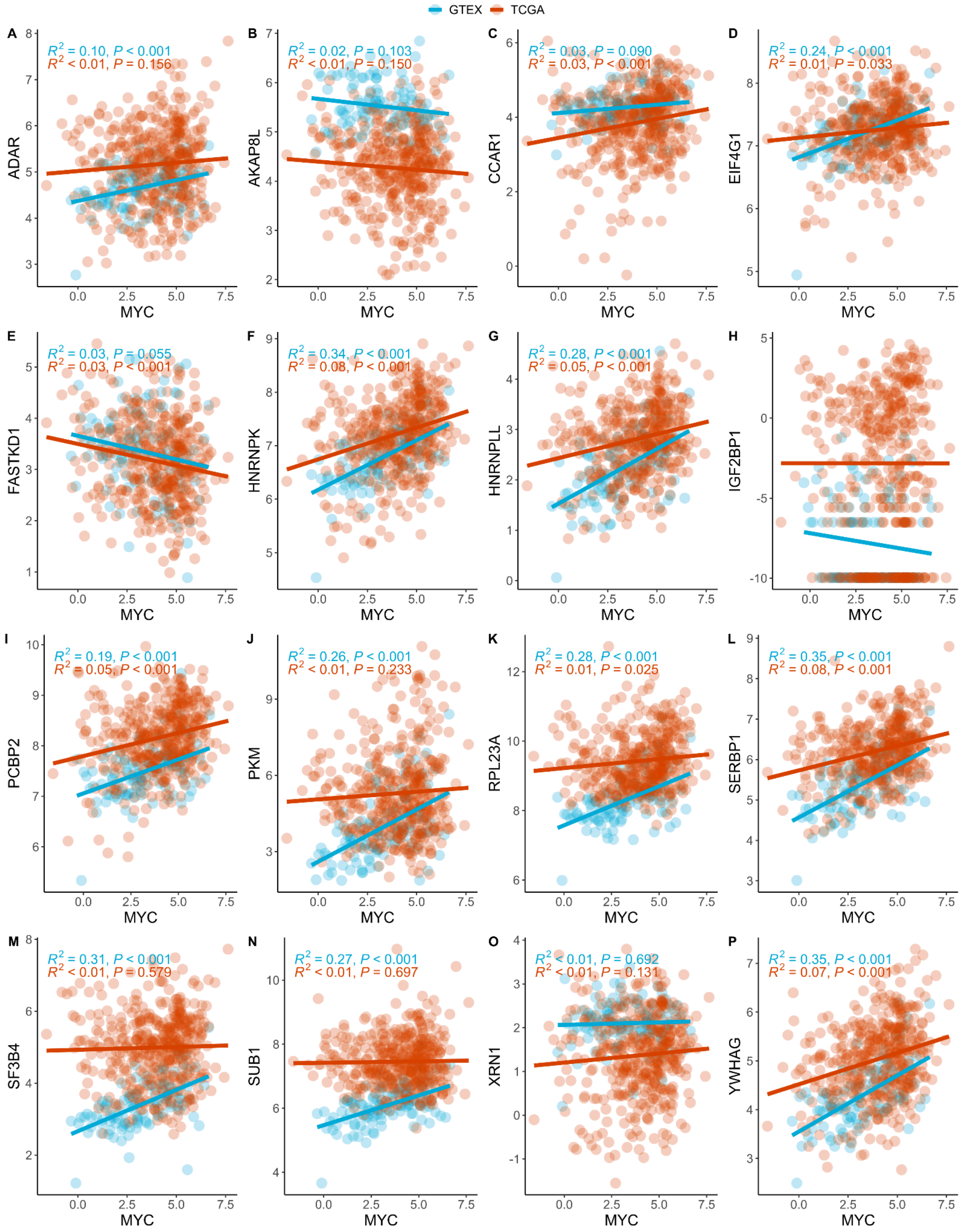

Figure S9. Co-expression changes between LIHC and GTEx liver tissue for interactions selected based on RBPs are underscored in **Table 1** (MYC targets). Linear model fit is presented as solid lines together with its correlation coefficient R^2 and P value (*stat_cor* R function).

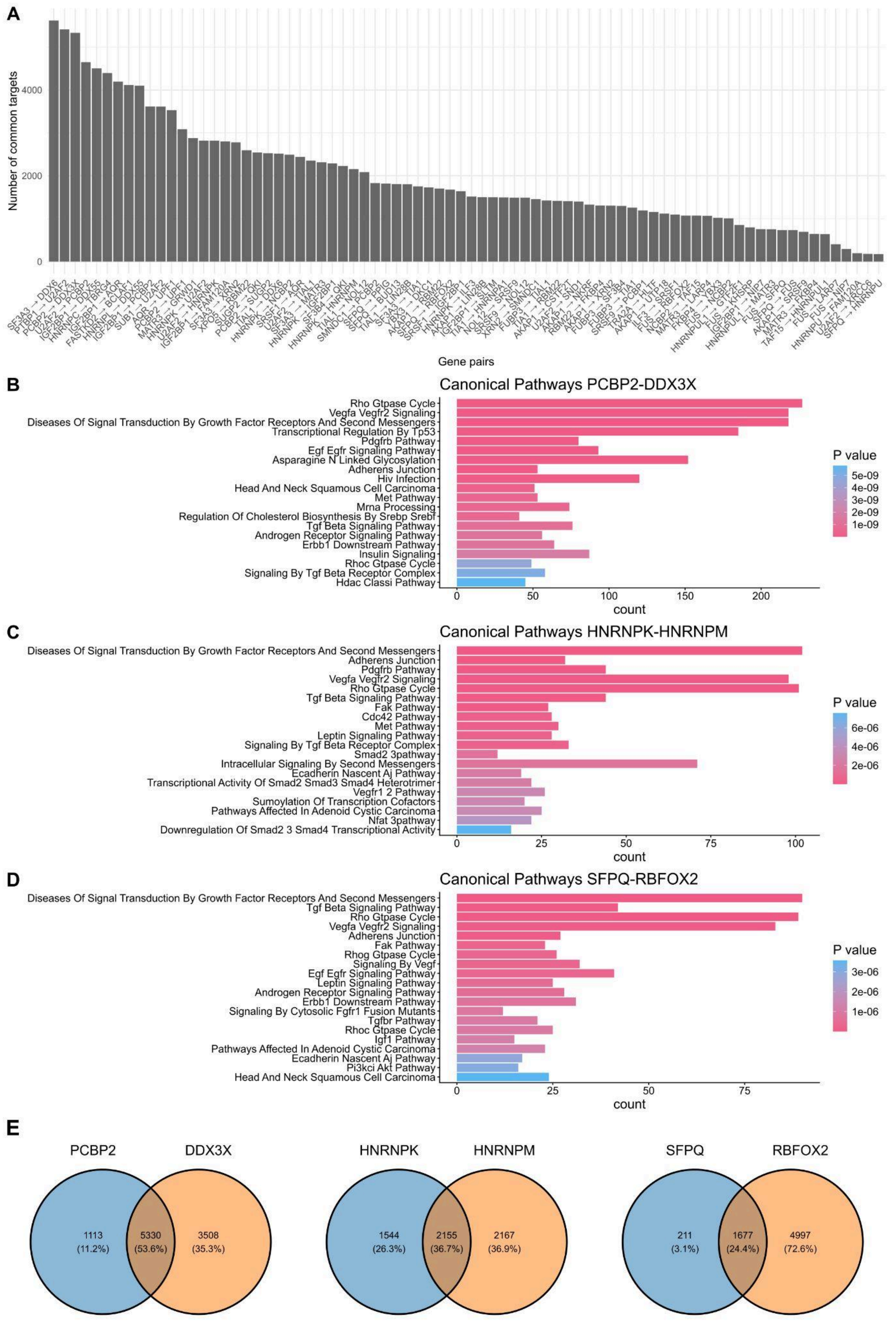

Figure S10. A. Number of common targets for all interaction **B-D.** The three most significant interactions were used to perform enrichment with canonical pathways for common targets of each RBP-RBP pair. Topmost 20 significant terms are displayed. **E.** Number of common targets for top three interactions.

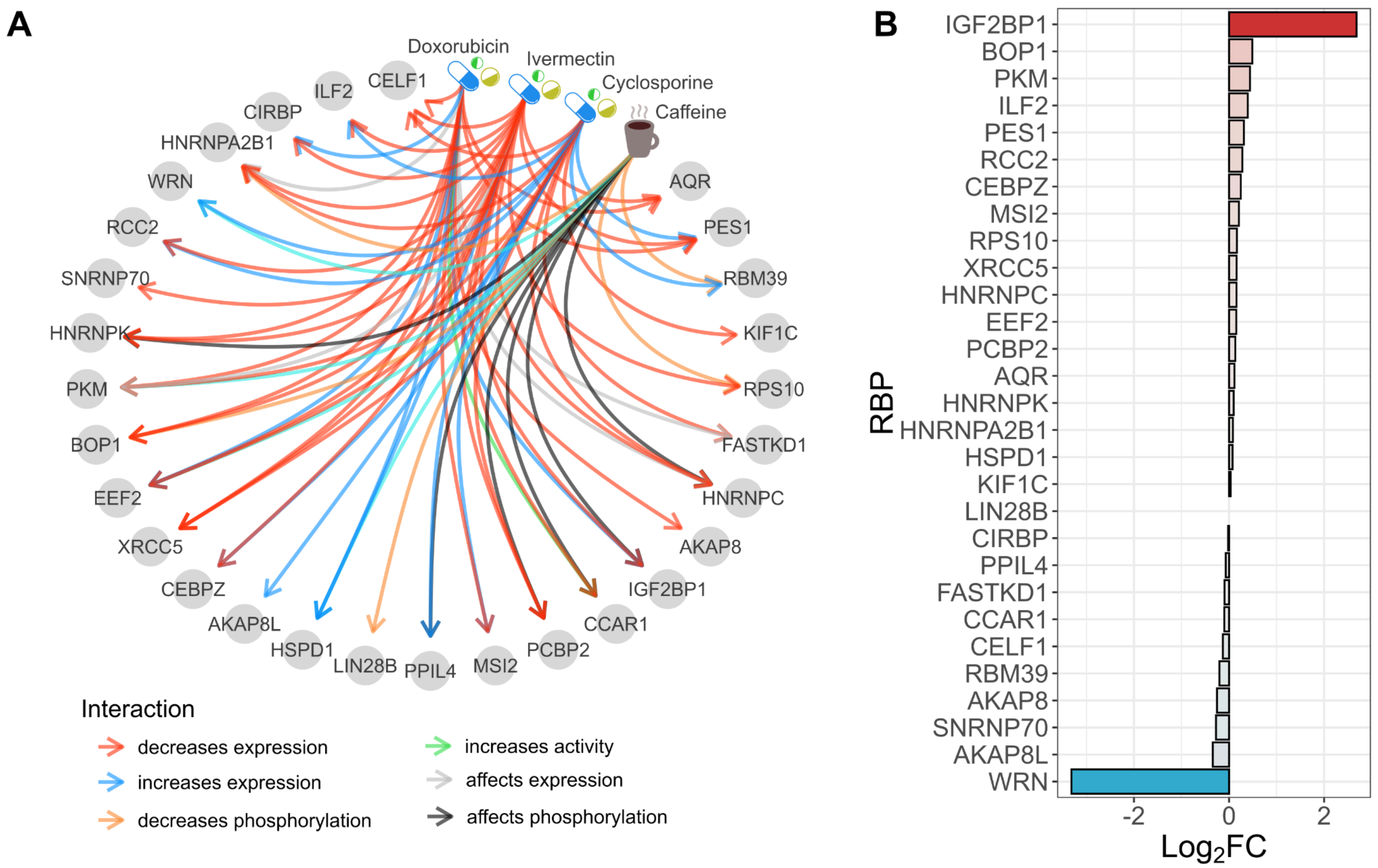

Figure S11. Drug repurposing and patients-based expression of noteworthy RBP-RBP interactions (**Table S5**). **A.** CTD-derived interactions between drugs and RBPs. The color of the edges represent various types of interactions. Drugs are marked as nodes with special icons. **B.** Log_2 fold-change (Log_2FC) based on expression from TCGA and GTEx cohorts. The colors of the bars represent the negative (blue) or positive (red) Log_2FC. Values were calculated as median RBP expression in LIHC over median RBP expression in healthy liver.

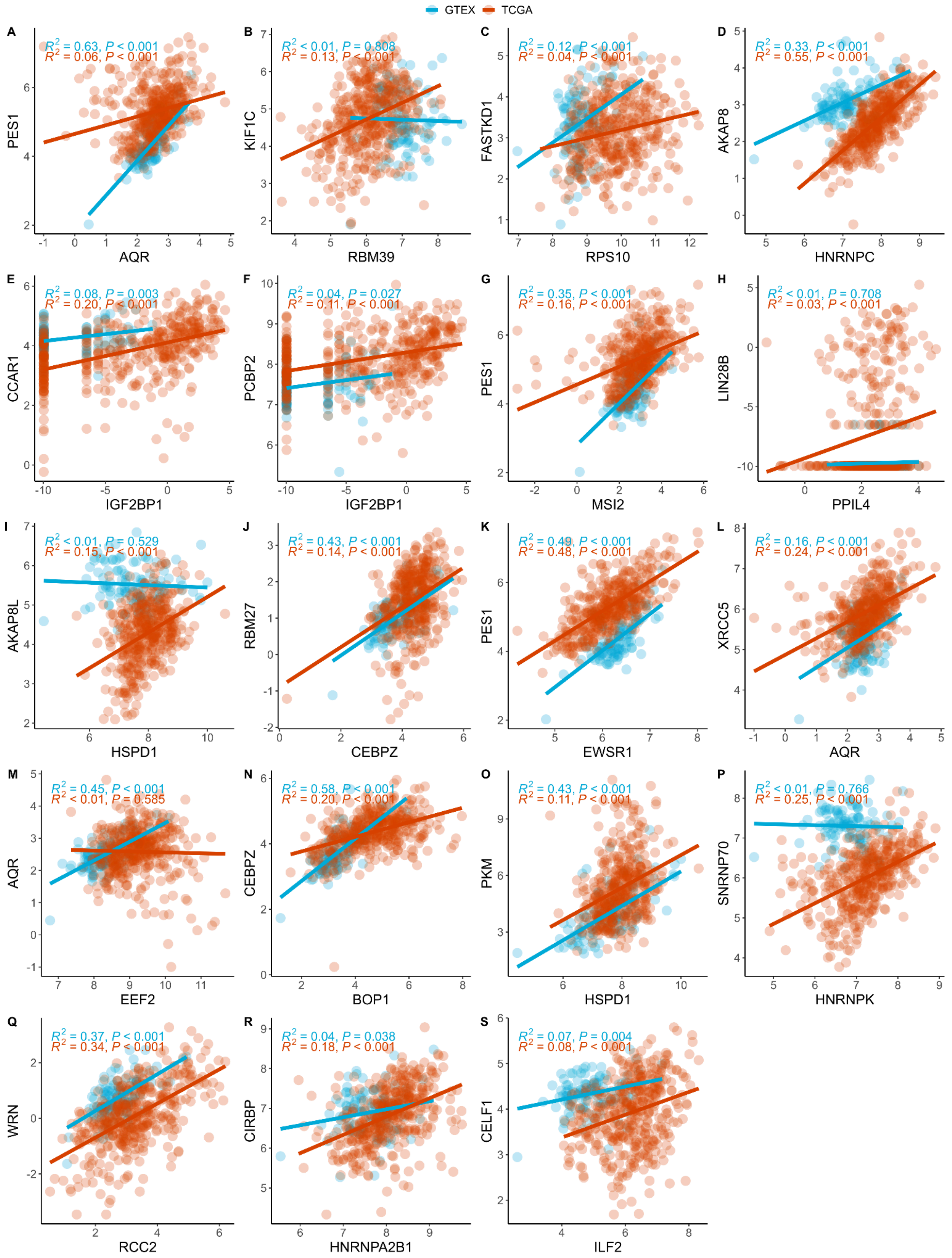

Figure S12. Co-expression changes between LIHC and GTEx liver tissue for interactions selected based on **Table S5**. Linear model fit is presented as solid lines together with its correlation coefficient R^2 and *P* value (*stat_cor* R function).

Supplementary tables

Table. S1 Adjacency matrix representing full consensus GRN for HepG2. The weights in the table represent consensus frequency. To extract the 3+ GRN, keep only links with an absolute frequency >= 0.3. Negative and positive weight indicates inhibition and activation, respectively. Direction should be read from columns to rows. (see Supplementary Files)

Table. S2 Adjacency matrix representing full consensus GRN for K562. The weights in the table represent consensus frequency. Negative and positive weight indicates inhibition and activation, respectively. Direction should be read from columns to rows. (see Supplementary Files)

Table. S3 Validation table of 3+ consensus GRN including all the validation features used in the analysis. (see Supplementary Files)

Table. S4 Information about databases used for validation. Last access 26th June 2024.

**Database**	**Version info**	**Link**
ENCODE	2	https://www.encodeproject.org/data/annotations/
UCSC Xena	-	https://xena.ucsc.edu/
EuRBPDB	v1 and v2	http://eurbpdb.gzsys.org.cn/
GRAND	1.5.4	https://grand.networkmedicine.org/
GRNdb	-	http://www.grndb.com/
FunCoup	5	https://funcoup.org/search/
MSigDB	v2023.2.Hs updated October 2023	https://www.gsea-msigdb.org/gsea/msigdb/
CLUEreg	GRAND (1.5.5)	https://grand.networkmedicine.org/analysis/
CTD	April 2024	https://ctdbase.org/

Table. S5 A summary of the most noteworthy predicted interactions between RBPs in liver cancer based on several factors concerning benchmarking and validation.

**Interaction**	**Type of interaction**	**Group**
AQR→PES1	inhibition	The highest chance of being true positive based on consensus benchmark (consensus frequency ≥ 0.7)
RBM39→KIF1C	inhibition	
RPS10→FASTKD1	activation	
HNRNPC→AKAP8	activation	Confirmed with eCLIP-seq and RAPseq
IGF2BP1→CCAR1	activation	
IGF2BP1→PCBP2	activation	
MSI2→PES1	activation	Top three interactions based on validation score
PPIL4→LIN28B	activation	
HSPD1→AKAP8L	activation	
CEBPZ→RBM27	activation	The highest validated TF with GRAND and GRNdb
AQR→XRCC5	activation	Both genes in the DNA repair pathway based on community enrichment analysis
EEF2→AQR	inhibition	The highest negative Log_2 co-expression fold-change
BOP1→CEBPZ	activation	The lowest number of literature mentions
HSPD1→PKM	activation	The highest number of literature mentions
HNRNPK→SNRNP70	activation	The highest positive Log_2 co-expression fold-change
RCC2→WRN	activation	The lowest *P* value indicating an impact on overall survival
HNRNPA2B1→CIRBP	activation	
ILF2→CELF1	activation	

Table S6. Drug repurposing was performed with CLUEreg on selected sets of targets. We marked the source of gene expression (GE) used for a given target list. The description includes a typical target of a given drug.

Targets	Drug	Cosine similarity	Tau	Effect	GE	Description
GRN 5+	Irinotecan	0.0778	0.01	Similar	GRN-based	DNA topoisomerase I inhibitor [1]
	SKF-89976A	0.0669	0.2268	Similar	GRN-based	GABA transporter 1 inhibitor [2]
IGF2BP1 top 20% targets from eCLIP-seq and RAPseq	Garcinol	-0.1401	0.0	Reverse	TCGA vs GTEx	inhibits STAT3 activation [3]
	Gallic-acid	-0.1398	0.0008	Reverse	TCGA vs GTEx	inhibits proliferation by arresting cells at the G2/M phase [4]
AQR targets top 20% DEGs in LIHC 2+ GRN	IWP-2	-0.1291	0	Reverse	TCGA vs GTEx	Inhibitor of Wnt production [5]
	Foxy-5	-0.1203	0.0101	Reverse	TCGA vs GTEx	WNT5A-mimicking peptide [6]
	Purpurogalin	-0.1213	0.0075	Reverse	TCGA vs GTEx	MEK1/2 inhibitor [7]
U2AF2 targets top 20% DEGs in LIHC 2+ GRN	Foxy-5	-0.1002	0	Reverse	TCGA vs GTEx	WNT5A-mimicking peptide [6]
	IWP-2	-0.0902	0.0002	Reverse	TCGA vs GTEx	Inhibitor of Wnt production [5]
	FH-535	-0.0785	0	Reverse	TCGA vs GTEx	Inhibits WNT pathway [8]

Supplementary references

1. Gilbert, D. C., Chalmers, A. J. & El-Khamisy, S. F. Topoisomerase I inhibition in colorectal cancer: biomarkers and therapeutic targets. *Br. J. Cancer* **106**, 18–24 (2012).
2. Moldavan, M., Cravetchi, O. & Allen, C. N. GABA transporters regulate tonic and synaptic GABAA receptor-mediated currents in the suprachiasmatic nucleus neurons. *J. Neurophysiol.* **118**, 3092–3106 (2017).
3. Sethi, G. *et al.* Inhibition of STAT3 dimerization and acetylation by garcinol suppresses the growth of human hepatocellular carcinoma in vitro and in vivo. *Mol. Cancer* **13**, 66 (2014).
4. Jiang, Y. *et al.* Gallic Acid: A Potential Anti-Cancer Agent. *Chin. J. Integr. Med.* **28**, 661–671 (2022).
5. García-Reyes, B. *et al.* Discovery of Inhibitor of Wnt Production 2 (IWP-2) and Related Compounds As Selective ATP-Competitive Inhibitors of Casein Kinase 1 (CK1) δ/ε. *J. Med. Chem.* **61**, 4087–4102 (2018).
6. Kelsey, R. Foxy–5 in prostate cancer model. *Nat. Rev. Urol.* **14**, 638–638 (2017).
7. Xie, X. *et al.* Purpurogallin is a novel mitogen-activated protein kinase kinase 1/2 inhibitor that suppresses esophageal squamous cell carcinoma growth in vitro and in vivo. *Mol. Carcinog.* **58**, 1248–1259 (2019).
8. Galuppo, R. *et al.* Synergistic inhibition of HCC and liver cancer stem cell proliferation by targeting RAS/RAF/MAPK and WNT/β-catenin pathways. *Anticancer Res.* **34**, 1709–1713 (2014).
